# A Protein Complex Map of *Trypanosoma brucei*

**DOI:** 10.1371/journal.pntd.0004533

**Published:** 2016-03-18

**Authors:** Vahid H. Gazestani, Najmeh Nikpour, Vaibhav Mehta, Hamed S. Najafabadi, Houtan Moshiri, Armando Jardim, Reza Salavati

**Affiliations:** 1 Institute of Parasitology, McGill University, Ste. Anne de Bellevue, Quebec, Canada; 2 Department of Biochemistry, McGill University, Montreal, Quebec, Canada; 3 McGill Centre for Bioinformatics, McGill University, Montreal, Quebec, Canada; 4 Centre for Host-Parasite Interactions, Institute of Parasitology, McGill University, Ste. Anne de Bellevue, Quebec, Canada; New York University School of Medicine, UNITED STATES

## Abstract

The functions of the majority of trypanosomatid-specific proteins are unknown, hindering our understanding of the biology and pathogenesis of *Trypanosomatida*. While protein-protein interactions are highly informative about protein function, a global map of protein interactions and complexes is still lacking for these important human parasites. Here, benefiting from in-depth biochemical fractionation, we systematically interrogated the co-complex interactions of more than 3354 protein groups in procyclic life stage of *Trypanosoma brucei*, the protozoan parasite responsible for human African trypanosomiasis. Using a rigorous methodology, our analysis led to identification of 128 high-confidence complexes encompassing 716 protein groups, including 635 protein groups that lacked experimental annotation. These complexes correlate well with known pathways as well as for proteins co-expressed across the *T*. *brucei* life cycle, and provide potential functions for a large number of previously uncharacterized proteins. We validated the functions of several novel proteins associated with the RNA-editing machinery, identifying a candidate potentially involved in the mitochondrial post-transcriptional regulation of *T*. *brucei*. Our data provide an unprecedented view of the protein complex map of *T*. *brucei*, and serve as a reliable resource for further characterization of trypanosomatid proteins. The presented results in this study are available at: www.TrypsNetDB.org.

## Author Summary

Due to high evolutionary divergence of trypanosomatid pathogens from other eukaryotes, accurate prediction of functional roles for most of their proteins is not feasible based on homology-based approaches. Although protein co-complex maps provide a compelling tool for the functional annotation of proteins, as subunits of a complex are expected to be involved in similar biological processes, the current knowledge about these maps is still rudimentary. Here, we systematically examined the protein co-complex membership of more than one third of *T*. *brucei* proteome using two orthogonal fractionation approaches. A high-confidence network of co-complex relationships predicts the network context of 866 proteins, including many hypothetical and experimentally unannotated proteins. To our knowledge, this study presents the largest proteomics-based interaction map of trypanosomatid parasites to date, providing a useful resource for formulating new biological hypothesises and further experimental leads.

## Introduction

The unicellular flagellated parasite *Trypanosoma brucei* is the causative agent of the fatal human African trypanosomiasis (HAT), also known as sleeping sickness, and the economically devastating disease Nagana in cattle. The life cycle of *T*. *brucei* comprise an insect stage (procyclic stage) and a mammalian stage. Trypanosomatid parasites, including *T*. *brucei*, are highly diverged from well-studied eukaryotes such as yeast and mammals [1–3], resulting in a lack of sequence similarity with model organisms for the majority of their proteins. This has led to inapplicability of homology-based approaches for identification of potential functions of more than half of all trypanosomatid genes [4].

Protein interaction maps offer an invaluable resource for functional annotation of proteins [5]. Current methodological/instrumental advances have led to the development of several *ex vivo*, *in vivo*, and *in silico* approaches to systematically chart protein interactions and complexes [6]. Optimized yeast two-hybrid (Y2H) approaches have been employed to infer pairwise interactions among proteins [7, 8]. Immunoprecipitation [9], biochemical fractionation [10–13], and affinity purification (AP)-based approaches [14–16] are widely used for the identification of protein complexes in a specific cell context. Additionally, functional association of proteins can be predicted computationally using data types such as transcriptomics data [17], synthetic lethality [18], and chemical sensitivity [19]. However, each of these approaches has limitations and is inherently associated, in varying degrees, with false positive and negative results. In AP-based approaches, for example, tagging the protein may affect the binding partners of the tagged protein by inactivation, capping the binding site, or changing the localization of the protein. Highly expressed proteins are also often co-purified with the tagged protein as a false positive contaminant. Moreover, transient interactions are likely to be lost if stringent conditions are used for the purification of the tagged protein. In biochemical fractionation strategies, fortuitous interactions can arise because confounding protein complexes can still be present in the same fraction regardless of in-depth fractionation [10]. In addition to non-negligible false positive rates, the Y2H system is relatively weak at detection of co-complex associations, although it works well at capturing binary, particularly transient, interactions [20]. Therefore, the integration of data from different approaches has been shown to improve the precision of protein maps [21].

To explore the protein complexes underlying the survival and pathogenesis of *T*. *brucei*, we performed four high resolution fractionation experiments benefiting from two orthogonal, complementary biochemical approaches. High-resolution mass spectrometry analysis of the fractions led to the construction of a global co-fractionation network for *T*. *brucei* in procyclic life stage. Evaluation of the constructed network demonstrated that it has topological and biological characteristics that are similar to those observed in the sampled networks of model organisms from previous large-scale studies. Importantly, our results demonstrated significantly higher precision for those interactions that were supported by the two orthogonal fractionation approaches compared to those that co-fractionated only in one approach. To extract a high-confidence core network, we combined the fractionation-derived network with other orthogonal resources of protein-protein interaction data. This high-confidence network predicts the network context of 866 protein groups, including many hypothetical and experimentally unannotated proteins. Clustering of this high-confidence network led to the assignment of 716 protein groups to 128 complexes. To our knowledge, this study presents the largest proteomics-based interaction map of trypanosomatid parasites to date, providing a useful resource for formulating new biological hypothesises and further experimental leads. To showcase the utility of this protein complex map, we used it to reveal novel factors involved in the mitochondrial post-transcriptional regulation of *T*. *brucei*, and validated them by several independent experiments.

## Materials and Methods

### Whole cell protein extract preparation

Late log phase (~2 x 10^7^ cells/ml) *T*. *brucei* procyclic form IsTaR 1.7 A cells (a derivative of EATRO 164) were grown in 225 cm^2^ flasks to obtain 4×10^9^ cells. The cells were harvested by centrifugation at 6000 x g for 10 min at 4°C, and washed once with ice-cold glucose-supplemented PBS (6mM glucose). The cells were then resuspended in 500 μl lysis buffer [10 mM Tris-HCl pH 7.2, 10 mM MgCl_2_, 100 mM KCl, 1 μg/ml pepstatin, 1 mM DTT, 1% triton X-100 and 1 x EDTA-free protease inhibitor cocktail (Roche)] and incubated on a tube rotator for 15 min at 4°C. The lysate was treated with 40 units of RNase-free DNase I (Roche) for 1 h on ice and cleared twice by centrifugation at 16,000x g for 15 min at 4°C.

### Cytosolic and mitochondrial protein extract preparation

The methods for preparing the extracts were adapted from conventional purification techniques [22–24]. Cell pellets were washed with ice-cold glucose-supplemented PBS as above, resuspended in 30 ml DTE buffer [1 mM Tris-HCl pH 8.0 and 1 mM EDTA] containing a protease inhibitor cocktail (Roche), and lysed using a 40 ml sterile tight-fitting Dounce-homogenizer on ice. The lysate was immediately made isotonic by adding a 2 M sucrose stock solution to a final concentration of 250 mM, and the mitochondria were centrifuged at 15,800 x g for 10 min at 4°C. The mitochondrial pellet was treated with 0.3 mM CaCl_2_ and 40 Us of RNase-free DNase I (Roche) in 4.6 ml STM buffer [20 mM Tris-HCl pH 8.0, 250 mM sucrose and 2 mM MgCl_2_] for 1 hr on ice and precipitated again. The mitochondrial lysate was prepared in 500 μl lysis buffer as described above for whole cells. The supernatant obtained upon collecting mitochondria at 15,800 x g, represents a crude preparation of the cytosol. This was centrifuged at 100,000 x g for 1 h at 4°C to eliminate small organelle contamination.

### Glycerol gradient fractionation and protein identification

Whole cell and mitochondrial extracts were resolved on 10–30% linear glycerol gradients [40 mM HEPES pH 7.9, 20 mM Mg(OAc)_2_, 100 mM KCl, and 2 mM EDTA] at 178,000x g for 6 hr at 4°C, and fractionated into 46–48 fractions (250 μl each), as described elsewhere [25]. Protein separation on these gradients were standardized with known amounts of BSA (Bovine serum albumin), catalase and IgM (Immunoglobulin M), with apparent masses of 66 KDa, 230 KDa and 970 KDa, respectively.

The glycerol gradient fractions were analyzed using an Agilent 1100 series capillary LC system and a ThermoScientific LTQ linear ion trap mass spectrometer. Fifty μl portions of each fraction were concentrated to approximately 10 μl volume by vacuum centrifugation, and 30 μl of 8M urea, 1.0 M Tris (pH 8.5), 8 mM CaCl_2_, and 0.2 M methylamine added. Cysteines were then reduced by 14 mM dithiothreitol (DTT) at 50°C for 15 min, and alkylated by 33 mM iodoacetamide at room temperature for 30 min. The urea concentration in the samples was then diluted to 2M concentration and trypsin (Sigma, T6567) added at an approximate ratio of 1:25 enzyme:substrate during an overnight digestion. Digests were then acidified by addition of formic acid at a final 5% concentration, vacuum concentrated to approximately 25 μl and injected for LC-MS/MS analysis. Electrospray ionization was performed with no sheath gas using an ion max source fitted with a 34 gauge metal needle. Samples were applied at 20 μl/min to a trap cartridge (Michrom BioResources, Auburn, California, USA) and then switched onto a 0.5×250 mm Zorbax SB-C18 column (Agilent) using a mobile phase containing 0.1% formic acid and a 7–30% acetonitrile (ACN) gradient over 95 min, with a 10 μl/min flow rate.

Peptide sequences were determined by comparing the observed MS/MS spectra to theoretical MS/MS spectra of peptides generated from a *T*. *brucei* protein database containing 9,826 entries (www.tritrypdb.org, release 3.2) comprised of both forward and reversed sequences using the program Sequest (Version 27, rev. 12, ThermoScientific). SEQUEST was configured as follows: parent ion average mass tolerance of 2.5 Da, fragment ion monoisotopic mass tolerance of 1.0 Da, tryptic enzymatic cleavage, a maximum of 2 missed cleavages, static C+57 modifications, and variable M+16 modifications. Peptides were filtered at 2% false discovery rate (FDR) for whole cell cytosolic samples and 5% FDR for mitochondrial samples using the PAW pipeline [26] and the target/decoy method. It should be noted that the reported peptide-to-spectrum match (PSM) FDRs are for the entire set of fractions in each experiment; and the FDR per fraction is much less than 1% even for the mitochondrial glycerol gradient fractionation data.

During the protein identification, we created protein groups by grouping the paralogous proteins (proteins that were generated by duplication events within the genome) with nearly identical protein sequences together. The total number of protein groups identified in this study was 1526, using a minimum of 2 unique peptides per protein, at a false discovery rate of about 1% for the combined fraction analysis and about 3% for the individual fraction analysis based on comparison of hits identified for forward and reverse sequences. Protein abundances were based on MS2 intensity-weighted spectral counting, which have been shown to provide accurate protein abundance estimates from LTQ linear ion trap mass spectrometer [27].

### Ion exchange chromatography fractionation, protein identification, and quantitation

Cytosolic and mitochondrial extracts were resolved by liquid chromatography using tandem cationic (S)–anionic (Q) exchange columns (UNOsphere, Biorad), adapted from [10, 28]. The mobile phase consisted of buffer A [10 mM Tris-HCl pH 7.8, 10 mM MgCl_2_, 50 mM KCl and 1 mM DTT] and buffer B [buffer A + 950 mM KCl]. Chromatography was performed using Beckman-Coulter Gold high performance liquid chromatography system. Samples were passed through a 0.22 μm membrane, loaded on the columns and then washed for 15 mins with buffer A, followed by 0–50% buffer B (1%/min), a 10 min wash and then 50–100% buffer B (5%/min). The flow-rate was maintained at 400 μl/min, and approximately 40 fractions (800 μl each) were collected.

The ion exchange separated proteins were analyzed using a Dionex NCP-3200RS UltiMate RSLCnano UPLC system and Thermo Scientific Orbitrap Fusion mass spectrometer. Pairs of adjacent fractions were pooled to create approximately 20 final fractions from each cytosol and mitochondria preparation and up to 200 μl of each were dried by vacuum concentration. Samples were then digested by trypsin (Sigma, T6567) as described above, except 50 μl of urea digestion buffer and an enzyme:substrate ratio of 1:10 was used. Following vacuum centrifugation to dryness, samples were dissolved in up to 100 μl of 5% formic acid, centrifuged at 16,000 x g for 10 min to remove particulates, and approximately 2 μg of each digest injected onto an Acclaim PepMap 100 μm x 2 cm NanoViper C18, 5 μm peptide trap on a switching valve. After 10 min of loading at 5μl/min, the trap column was switched on-line to a PepMap RSLC C18, 2 μm, 75 μm x 25 cm EasySpray column (Thermo Scientific). Peptides were then separated using a 7.5–30% ACN gradient over 60 min in mobile phase containing 0.1% formic acid at a 300 nl/min flow rate and ionized using an EasySpray NanoSource (Thermo Scientific). Survey scans were performed by the Orbitrap mass analyzer at a resolution of 120,000, and data-dependent MS2 scans acquired by the linear ion trap in rapid mode. Data-dependant scanning used a 30 sec exclusion time, repeat count of 1, exclusion of +1 charge ions, top speed mode, and a 3 sec dwell time between survey scans.

MaxQuant (version 1.5.2.8) was used for peptide and protein identification from Orbitrap Fusion data. MaxQuant was configured as follows: FASTA database was a *T*. *brucei* protein database containing 11,567 entries (www.tritrypdb.org, release 24.0), default decoy sequence generation, tryptic enzymatic cleavage, default parent ion and fragment ion mass tolerances, a maximum of 2 missed cleavages, static C+57.02 modifications, variable M+15.995 modifications, and target peptide FDR of 1% (cytosolic) or 2% (mitochondrial). A high protein FDR target was chosen because MaxQuant does not compute protein FDR per sample in multi-sample experiment designs; per sample protein FDRs are much less than overall experiment-wide protein FDR when there are many samples. Protein quantification used the protein total intensity values in the MaxQuant protein summary files. We also invoked a two peptide per protein per sample (one LC run) criteria to reduce protein identification noise.

### *T*. *brucei* cell culture, RNA interference, and growth curves

The RNA interference (RNAi) vector was prepared by amplifying 433 bp fragment from the open reading frame of Tb927.10.7910 gene. The amplified fragment was introduced into p2T7-177 [29]. Linearized plasmids were transfected into procyclic form 29–13 cells by electroporation, and transfected cells were selected by adding 2.5 μg/ml pheleomycin. Growth effect in RNAi cell lines was monitored for up to 10 days in the presence and absence of 2.5 μg/ml tetracyclin.

### Quantitative real time PCR

RNA was collected from uninduced and Tet-induced RNAi Tb927.10.7910 cell line at day-3 post induction, selected based on the observed growth defect. RNA was isolated from ~10^8^ cells using TRIzol (Invitrogen). Ten micrograms of total RNA were treated with DNA-*free* DNase Kit (Ambion) to remove any residual DNA. Purified RNA was reverse transcribed in 25 μl RT-PCR reactions including TagMan reverse transcriptase and random hexamer primers using TagMan Reverse Transcription Kit (Applied Biosciences). Different RT-PCR reactions were performed with primers specific to pre-edited, edited, and never-edited mitochondrial transcripts, as described previously [30] and primers that flank the junctions of the adjacent genes, 9S/ND8, Cyb/A6, and RPS12/ND5 [31] using a Corbett rotor gene 3000. Data were normalized against the 18S rRNA as an internal reference, and were confirmed for several genes considering the β-tubulin as the internal reference. Each mRNA target was analyzed in two biological replicates and three technical replicates. Relative changes in RNA abundance were calculated using ΔΔCt, as described before [32]. The sequence information for primers used in this study is provided in S1 Table.

### Tagged cell line construction and immunofluorescence assays

To generate C-myc-tagged cell lines, the open reading frames of Tb927.1.1730, Tb927.10.1730, and Tb927.10.7910 were amplified and cloned into *Bam*HI and *Hpa*I sites (for Tb927.1.1730) or *Bam*HI and *Hind*III sites (for Tb927.10.1730 and Tb927.10.7910) of pHD-1700 [33]. All constructs were digested with *Not*I before transfection into 13–13 procyclic form cells.

C-myc tagged cell lines were induced with 0.5 μg/ml tetracycline for 48 hr and then used to prepare slides for immunofluorescence analysis. Briefly, mid-log phase cells were fixed with 4% paraformaldehyde in PBS and placed on poly-L-lysine-treated round coverslips (Fisher). Cells were permeabilized with 0.2% Triton-X-100 in PBS and blocked with 3% BSA in PBS. Anti-myc antibody (1:500) was used to visualize myc-tagged cells. Mitotracker (Invitrogen) was used to stain the organelles and DAPI was used for DNA staining. Slides were analyzed using a Nikon up-right microscope.

### Mitochondrial extract preparation and immunoprecipitation

Hypotonic purification of mitochondria from ~5 x 10^7^ uninduced and tet-induced PF cmyc-Tb927.1.1730, cmyc-Tb927.10.1730, and cmyc-Tb927.10.7910 cells was carried out as described above. The lysis buffer was prepared either with 40 U of RNaseOUT (Invitrogen) or 200μg/ml of RNase A from bovine pancreas (Sigma). Anti c-myc agarose conjugated beads (Sigma) were washed five times with 1ml ice-cold PBS at 4°C and subsequently washed once with 1ml of ice-cold immunoprecipitation wash buffer (Tris-HCL, pH 8.0, 10mM, NaCl 100mM, NP-40 0.1%, 1X complete EDTA-free protease inhibitor (Roch) and 1% PBS). After the last wash, 50 μl of beads were re-suspended for each reaction in 1 ml of ice-cold wash buffer and incubated for 1 hour at 4°C on a tube rotator. After adding the mitochondrial lysate to the beads, mixture was rotated for 2 hours at 4°C followed by centrifugation at 500rpm for 1 min at 4°C. After removing the supernatant (unbound proteins), the beads were washed four times with 1ml of immunoprecipitation wash buffer and then resuspended in SDS-PAGE loading dye. Aliquots of lysate, bound and unbound fractions were loaded on 10% SDS-PAGE gel. Proteins were transferred onto nitrocellulose membrane and probed with polyclonal antibodies against MRB 8170 and TbRGG2 (a generous gift from Laurie Read, state University of New York at Buffalo, USA).

### Construction of primary co-fractionation networks

Although the stoichiometric ratio among proteins that are involved in one complex is expected to be linear, this relationship can be more complicated for those participating in several complexes (As an example, see the fractionation patterns for proteins related to the RNA editing process presented in the validation section). For stringent analysis of data, we used a previously developed mutual information-based approach, termed context likelihood of relatedness (CLR) [34], to infer pairwise interactions among the proteins based on the observed fractionation patterns. In contrast to association measures such as Pearson correlation coefficient, mutual information is a measure of association that does not have strong prior assumption about the association type [35]. After calculating fractionation pattern similarity scores for each possible interaction using mutual information, CLR determines significant pairwise interactions by comparing the similarity score of each protein pair to a background joint distribution, which was obtained by the calculation of similarities for all possible interactions that each of the interacting proteins can be involved in. To construct the co-fractionation networks, we set the false discovery rate cut-off threshold at 0.05. False discovery rates were calculated using the provided functions in the CLR package [34]. However, since mutual information does not discriminate the type of association (positive or negative), we considered only those interactions as valid that their fractionation patterns were non-negatively correlated with each other as judged by Pearson correlation coefficient. Comparison of results for the mutual information-based version of CLR with the correlation-based version of this algorithm as well as conventional Pearson correlation coefficient-based method indicated that the former is more consistent with the previous knowledge on *T*. *brucei* protein complexes.

To assess whether the observed co-fractionation patterns for low abundant proteins is significant or unreliable, we generated 100 noisy datasets from each fractionation experiments. Expecting a uniform distribution of noises in the dataset, added noise to each cell in a dataset were modeled by Poisson distribution with the lambda equal to the value of the cell plus the noise term of [the lowest identified ion intensity in the dataset]/[No. of fractions in the dataset]. Those protein pairs that were significantly co-fractionated (FDR ≤0.05) in the original dataset, but lost their significant co-fractionation (p-value >0.05) in at least 10% of noisy datasets were discarded from the corresponding co-fractionation network.

### Modulation score

Modulation score examines the density of connections among a pre-specified group of nodes (e.g., proteins) in a given-network with the interaction density that is expected to occur by chance [36]. The density of interactions for a group of nodes is defined as the number of within-group interactions divided by the total number of interactions of the members of the group. This ratio is then compared to the density distribution generated over random groups with the same number of nodes in the network. In the original implementation of the modulation score, the density distribution was estimated by normal distribution assumption [36]; however, the distribution may not necessarily be normal. Therefore, we developed a modified version of the algorithm that uses a kernel smoothing function to estimate the distribution.

### Curation of high-confidence network

To find high-confidence interactions, we considered following available orthogonal resources that contain protein-protein interaction data:

#### STRING database

Interactions restricted to the proteins identified in this study with a medium confidence score (default setting in the STRING) were extracted from the database (version 10).

#### KEGG pathway

All pathways related to *T*. *brucei*-associated proteins were extracted from the KEGG database.

#### Interlog mapping

Although trypanosomatid organisms are highly diverged, they share some common processes and, consequently, common protein complexes with other well-studied eukaryotes. To map these conserved complexes, we first transferred protein interaction data from a highly conserved interactome of eukaryotic cells (see below) to *T*. *brucei* proteins. To remove those interactions that are not conserved in *T*. *brucei*, only the transferred interactions whose existence was also supported by our fractionation network (TbCF net) were considered as valid. To determine a highly conserved protein interaction map of eukaryotic organisms, we extracted the high confidence interaction networks (interaction scores above 0.9) of human and yeast from the STRING database. Using the orthologous groups defined by the InParanoid database [37], we extracted a sub-network that is common between these two networks. Considering the large evolutionary distance between yeast and humans, the created sub-network was highly enriched for the basic processes that are vital for eukaryotic cells. Next, the sub-network was mapped to *T*. *brucei* proteins based on the InParanoid database.

#### Literature search

Extensive literature searches were performed to find published interactions from small-scale studies of trypanosomatid proteins. In total, 24 studies constituting 81 experiments (including AP, immunoprecipitation, and Y2H experiment types) were considered (Only studies/experiments that provide interaction information for at least two proteins identified in this study was considered.). To construct interaction maps for experiments that contained protein complex information (e.g., AP and immunopercipitation), we considered a matrix model which assumes any two co-purified proteins can be directly connected to each other [38]. By restricting the network to proteins identified in this study, the preliminary literature-based network consisted of 13,307 interactions, containing both genuine and false positive interactions. Of these, 1421 interactions whose existence was also supported by the TbCF net, were considered as high-confidence.

#### Orthogonal reproducibility

Our analysis has suggested a high precision (estimated precision rate of 59%) for the orthogonally reproducible part of TbCF network (TbCF_OR_ net), the sub-network of TbCF net that were reproducible in both glycerol gradient and ion exchange high performance liquid chromatography experiments. Therefore, we considered as high confidence those protein pairs that were present in the TbCF_OR_ net, but there were not any orthogonal evidences about the interacting partners of at least one of the proteins in any of the four orthogonal evidences mentioned above.

### Estimation of precision and recall of the networks

To estimate the precision (percentage of interactions that are true) and recall (the fraction of overall true interactions that are present in the networks) of the constructed networks, benefiting from our extensive literature search, we created a gold standard set of >200 proteins in 20 distinct protein complexes (S3 Table), allowing us to evaluate our network against current literature. It should be noted that some of these complexes are partially identified and there might be some subunits that are still uncharacterized. We also ignored putative subunits that were suggested solely based on a single experiment like pull down without further experimental characterizations and/or verifications. The interactions between proteins in the same complex were defined as the positive set. Negative set were defined as interactions between proteins of different complexes. For more stringent analysis, we added to the negative set the other interactions that the subunits of the 20 complexes had with the rest of proteins in the network, unless they had external experimental evidences (e.g., pull down). Clearly, the defined negative set can contain some of genuine interactions that were not identified previously, leading to an underestimation of the calculated precision for the networks. Precision was defined as the number of interactions in a network that are in the positive set, divided by the total number of interactions in the network that belong to either positive or negative sets. For each network, two types of recalls were defined: 1) Recall for proteins: defined as the number of distinct subunits of the twenty complexes that are present in the network divided by the total number of known subunits identified in our mass spectrometry experiments; 2) Recall for the interactions: defined as the total number of interactions that the identified subunits of the same complex have in the network divided by the maximum number of interactions that they can have theoretically. Since in a network, the subunits of the same complex are often much sparser than the theoretically assumed fully-connected model, the calculated recalls for the interactions are usually small [10, 13].

### Network visualization and topological analysis

All networks were visualized using Cytoscape, a network visualization tool for Genome Space workflows [39]. NetworkAnalyzer, a plugin of Cytoscape, was used for the topological analysis of networks, including: node-degree distribution, shortest path-length distribution, and topological coefficient distribution [40].

### Measurement of semantic similarity between gene ontology terms

To examine the gene ontology-biological process similarity of interacting genes in the constructed networks, we used Resnik’s approach to quantify semantic similarity between gene ontology terms [41]. Semantic similarities between GO categories of biological processes and cellular compartments were calculated for each interacting protein pair present in a network, if both proteins were annotated in the uniprot database [42]. When calculating semantic similarities, we ignored those terms with evidence codes of NR (Not recorded), ND (No biological data available), and IEA (Inferred from Electronic Annotation). The GO-BP sematic similarities were calculated by GOssTO tool using default parameters [43].

### Statistical analysis

MATLAB R2014b software (The MathWorks Inc., Natick, MA) was used for the CLR score false discovery rate estimation and kernel-based p-value estimation. C# programing language was used for the calculation of the KEGG pathways modulation scores as well as construction of random networks. Other statistical analysis was performed using the R programing language.

### Accession numbers

The mass spectrometry data have been deposited to the ProteomeXchange Consortium (http://proteomecentral.proteomexchange.org) via the PRIDE partner repository [44] with the dataset identifier PXD002640. Full instrument settings, search parameters, pipeline processing details, and dataset statistics can be found in the PRIDE submission.

#### Id list for genes mentioned in the text

Tb927.1.1730, Tb927.1.3010, Tb927.10.10130, Tb927.10.10830, Tb927.10.11870, Tb927.10.1730, Tb927.10.5830, Tb927.10.7910, Tb927.11.16860, Tb927.11.7960, Tb927.2.6070, Tb927.5.3010, Tb927.6.1200, Tb927.8.8170.

## Results and Discussion

### Construction of the co-fractionation networks

**Fig 1:**
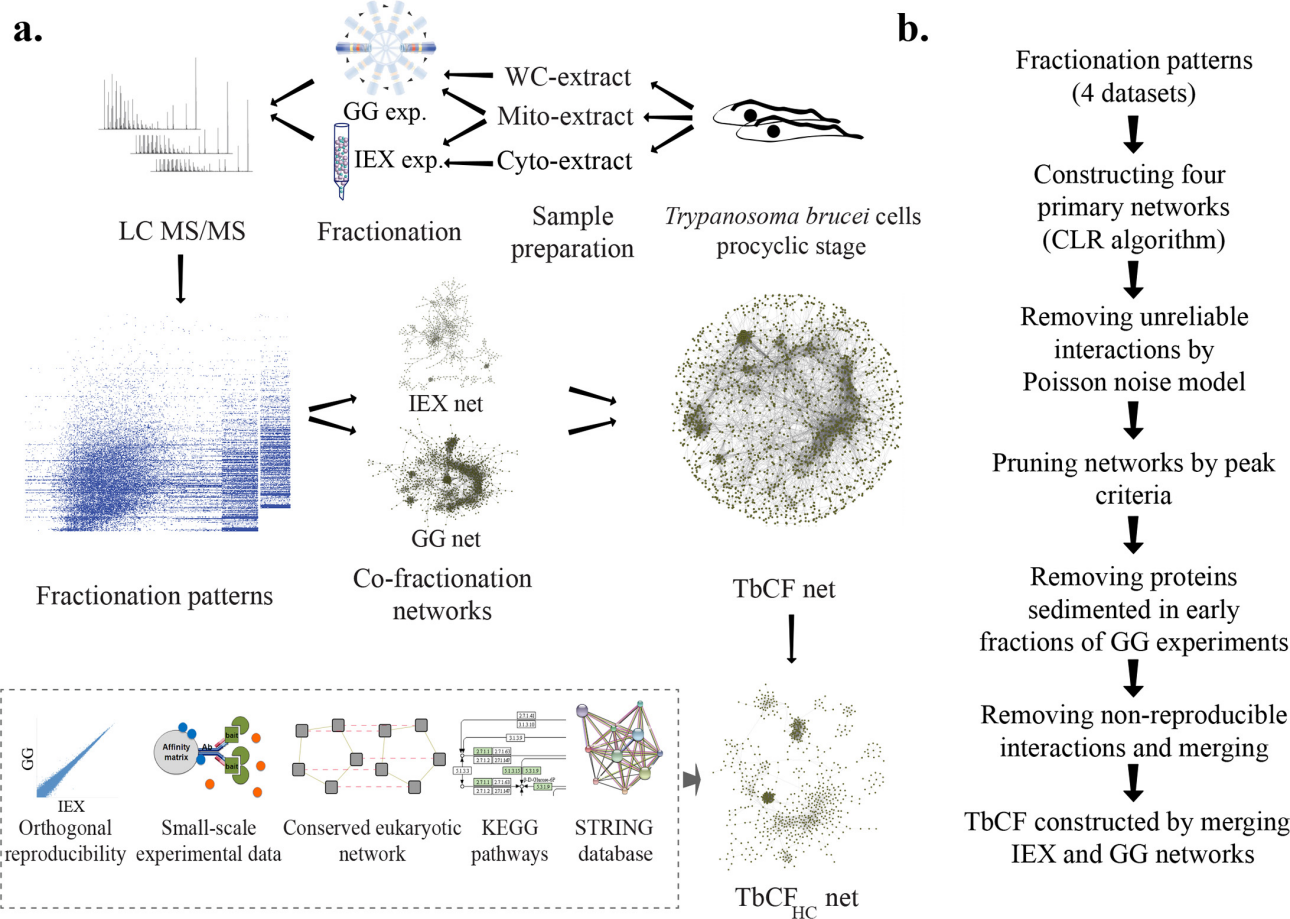
The strategy used for construction of a high-confidence protein interaction network. a) Preparations of cell extracts (whole-cell, and enriched extracts from mitochondria and cytosol) from *T*. *brucei* procyclic cells were subjected to in-depth fractionation using two different fractionation techniques. In total, 133 fractions were analyzed by mass spectrometry to generate the fractionation patterns for a total of 3354 *T*. *brucei* protein groups in four separate fractionation experiments. Next, a co-fractionation network for each of four experiments was constructed based on the observed fractionation patterns. After stringent filtration, these four networks were merged based on the employed separation technique to form GG and IEX networks. TbCF net was constructed by those interactions that were present either in IEX or GG networks. TbCF_HC_ net, the high-confidence subset of TbCF net, was generated by identification of interactions that were supported by at least one orthogonal resource. WC: Whole Cell; Mito: Mitochondrial; Cyto: Cytosolic; GG: Glycerol Gradient; IEX: Ion Exchange Chromatography. b) The computational pipeline used for the inference of TbCF net. Starting from the fractionation patterns, four preliminary co-fractionation networks were constructed and then refined with four additional filtration criteria to eliminate spurious interactions. First, those interactions that were sensitive to the addition of noise were eliminated. Second, the unshared-peak interactions were discarded from each co-fractionation network. Next, early co-sedimenting proteins (Proteins that are expected to not be involved stably in protein complexes) were eliminated from the networks. Finally, the non-reproducible interactions were removed and networks related to each fractionation approach were merged to generate GG and IEX networks. The GG co-sedimentation and IEX co-elution networks were merged together to form TbCF net.

**Fig 2:**
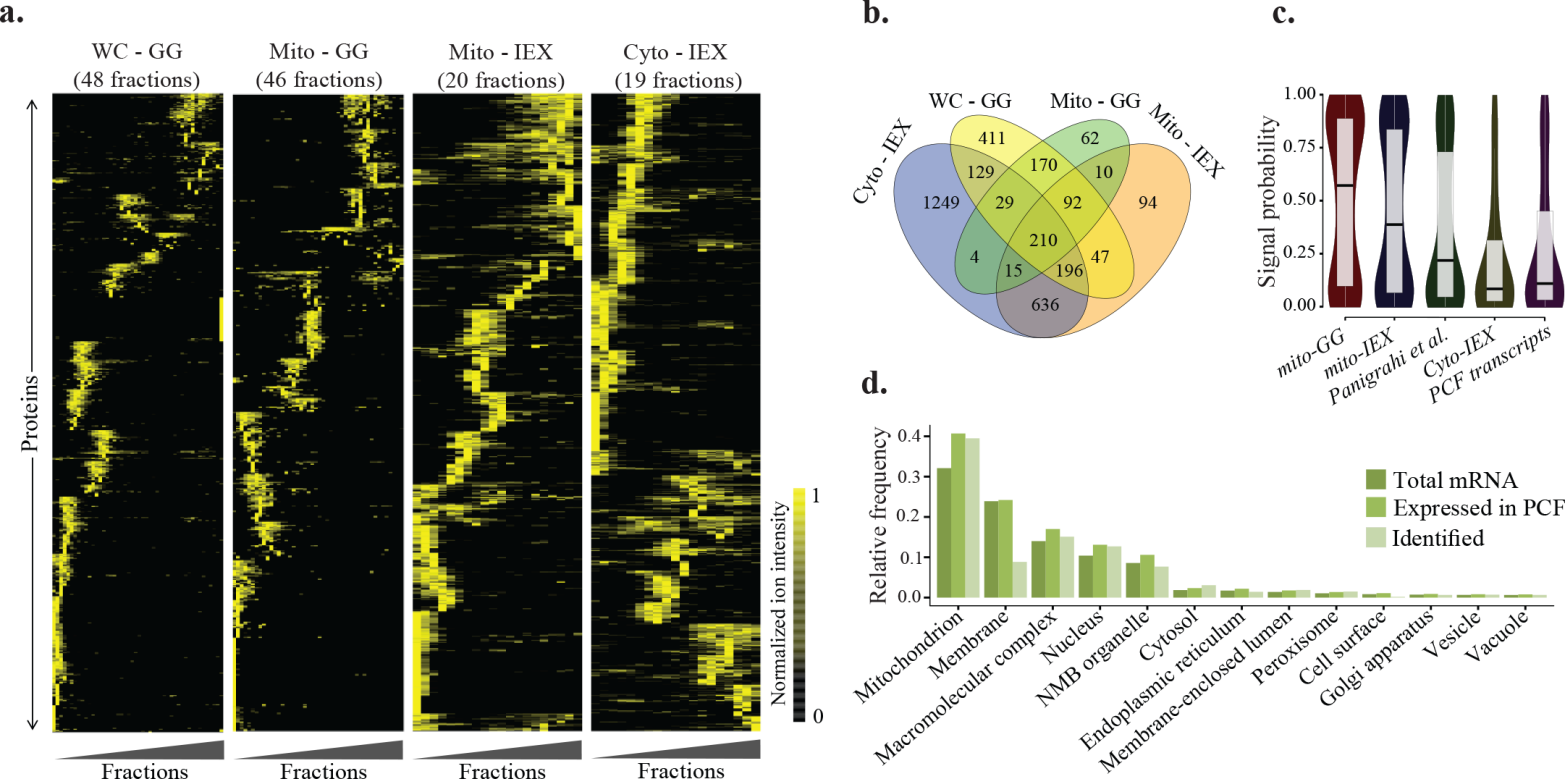
High resolution fractionation experiments on the procyclic stage of *T*. *brucei*. a) Hierarchical clustering of fractionation patterns for proteins identified in each of four experiments on *T*. *brucei* procyclic cells. Each row represents a protein and each column a fraction. It should be noted that the number of fractions as well as number of identified proteins varies among datasets (i.e., fractionation experiments). Moreover, datasets were analyzed independently and, therefore, the positions of proteins are not preserved in the graphs. b) Venn diagram representing proteins identified in each of four experiments. c) Comparison of the mitochondrial signal probability distribution for proteins identified in our mitochondrial and cytosolic enriched experiments with those identified by Panigrahi et al. [25] and total identified mRNAs in procyclic life stage of *T*. *brucei* [45]. The signal probability for each protein was calculated using the mitoProt web service [46]. As illustrated, the signal probability for our mitochondrial enriched samples were significantly higher (comparing to proteins identified in Panigrahi et al. [25]; p-value < 1.4E-08, Wilcoxon-Mann-Whitney rank sum test) and for the cytosolic experiment significantly lower (comparing to total procyclic transcripts; p-value <1.4E-07, Wilcoxon-Mann-Whitney rank sum test) than that of the other two groups. d) Gene ontology–cellular component (GO-CC) analysis of all identified proteins in this study compared with the mRNAs that are expected to be expressed in the procyclic stage [45] and total predicted mRNAs in *T*. *brucei*. NMB Organelle: Non-membrane-bounded Organelle.

Biochemical fractionation techniques have been widely applied to trypanosomatid organisms to test the possibility of physical associations among a set of pre-specified proteins. Fractionation approaches allow dissection of protein complexes based on different biochemical properties. In the glycerol gradient (GG) fractionation approach, protein complexes become separated according to their shape/density. Alternatively, complexes were fractionated on the basis of their overall charge in ion exchange high performance liquid chromatography (IEX) experiments. As summarized in Fig 1A, by coupling GG and IEX deep fractionation techniques with semi-quantitative, ultra-sensitive, mass spectrometry, we generalized the approach to chart a proteome-scale *T*. *brucei* interaction network. We were able to observe the fractionation pattern of 3354 protein groups (paralogous proteins with nearly identical sequences were grouped together) across total of 133 separate fractions from whole cell-GG (48 fractions), mitochondrial-GG (46 fractions), cytosolic-IEX (19 fractions), and mitochondrial-IEX (20 fractions) experiments on *T*. *brucei* procyclic form cells (Fig 2A). Due to complementary design of experiments, we were able to observe the fractionation patterns of 1398(42% of total) proteins in both GG and IEX fractionation approaches, providing a global picture of protein complexes present in *T*. *brucei* procyclic cells (Fig 2B).

Comparison of our results from mitochondrial experiments with a previously reported repository of mitochondrial proteins [25] revealed that 79% of the detected proteins in our experiments were independently supported to be present in the mitochondria. Comparing the mitochondrial signal probability of the proteins identified in our mitochondrial samples with the above mentioned list and also a list of transcripts that are expressed in procyclic life stage of *T*. *brucei* [45] suggested that the identified proteins were significantly enriched (comparing to proteins identified in [25]; p-value < 1.4E-08, Wilcoxon-Mann-Whitney rank sum test) for the mitochondrial proteins (Fig 2C). The same analysis on the proteins identified in the cytosolic experiment demonstrated a significant depletion (comparing to total procyclic transcripts; p-value <1.4E-07, Wilcoxon-Mann-Whitney rank sum test) of mitochondrial proteins in the sample. These results demonstrate the accuracy of the employed compartment enrichment procedures.

In total, proteins identified in our fractionation experiments cover 43% of all expressed protein coding genes in *T*. *brucei* procyclic stage [45] and 77–127% of number of proteins reported in previous proteome-wide SILAC studies [47–49]. To test for bias in the data, we examined the distribution of detected proteins in terms of cellular component and number of putative transmembrane domains. As expected, soluble proteins were preferentially detected by the employed fractionation approaches, while the membrane associated proteins were under-represented (Fig 2D and S1 Fig). The detected proteins did not show bias in terms of protein length (S1 Fig). Moreover, observing the transcriptome responses of the identified proteins in different life stages [50] and over the differentiation process [51, 52] indicated that they show over-expression trend in the procyclic life stage, but biased towards more abundant transcripts in the cell (S2 Fig). Because paralogous proteins with nearly identical sequences are expected to play similar functions in the cell, we randomly selected one representative protein from each protein group to construct a protein interaction map based on the observed fractionation patterns.

**Fig 3:**
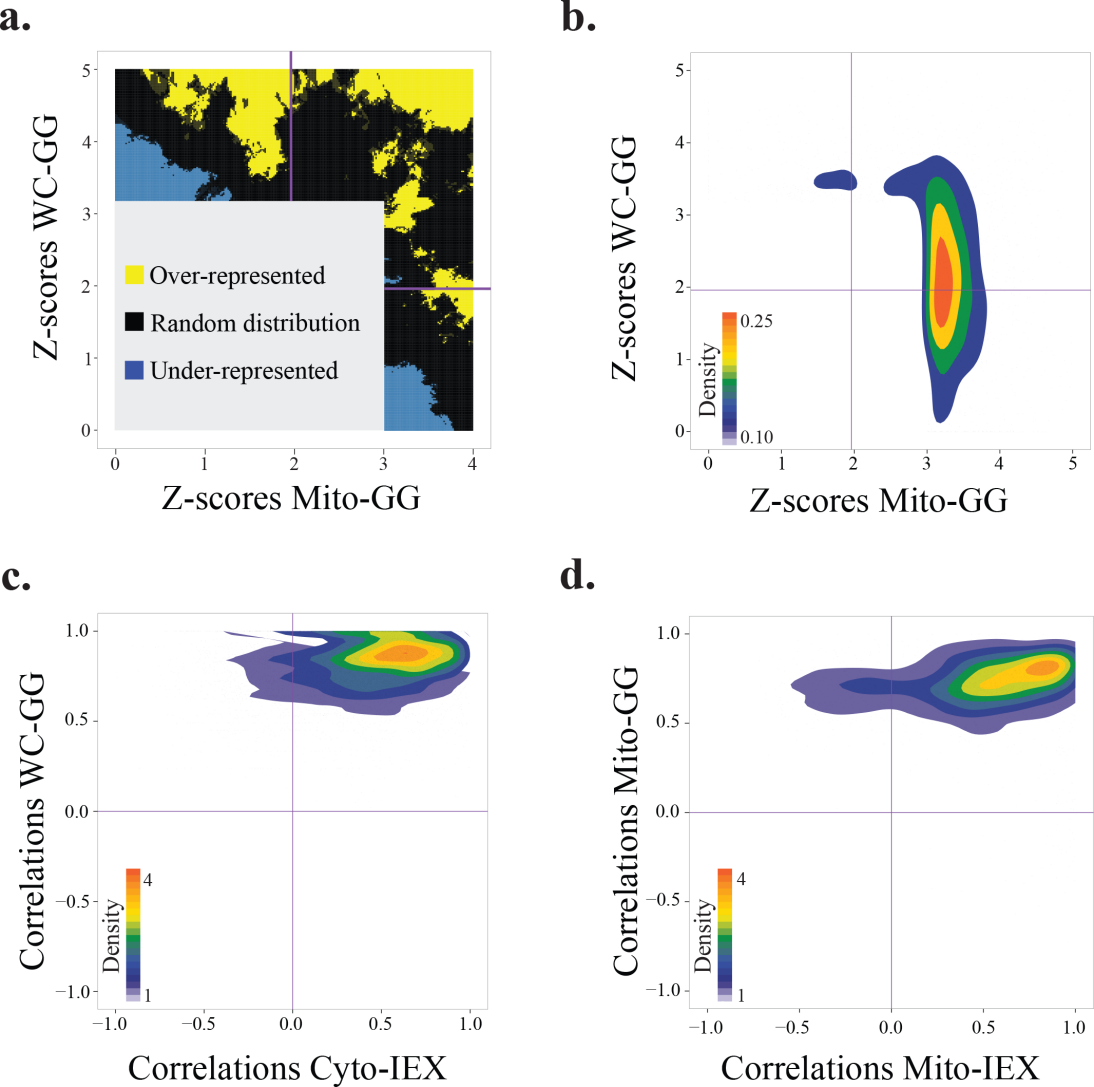
Validation of filtration steps used for stringent analysis of fractionation patterns. a) Interactions present in each of whole cell-GG and mitochondrial-GG networks were categorized as either shared-peak interactions (interacting proteins show a peak in at least one shared fraction) or unshared-peak interactions (interacting proteins show completely distinct peaks). Gray area represents the co-sedimentation space that was not significant in either fractionation experiment. Blue color represents the region depleted for the shared-peak interactions and the yellow region demonstrates the area for over-represented shared-peak interactions. As shown, the shared-peak interactions are highly depleted in non-reproducible regions (upper-left and lower-right regions in the graph). Enrichment at each point on the graph was calculated using a two-tailed hypergeometric test by focusing on the closest 110 interactions to that point (p-value ≤0.05). b) Distribution of z-scores for significant interactions identified in whole cell-GG and/or mitochondrial-GG experiments. The horizontal and vertical purple lines intersect with the XY-axes at points corresponding to the p-value equal to 0.05. As illustrated, more than half of interactions fall in the region that is significant in both experiments (upper-right region). c) Distribution of correlations for significant interactions identified in either whole cell-GG (WC-GG) or cytosolic-IEX (Cyto-IEX) datasets. As shown, interactions have clear peak (color coded in red) in the highly reproducible region (upper-right region). d) Distribution of correlations for significant interactions identified in either mitochondrial-GG (Mito-GG) or mitochondrial-IEX (Mito-IEX) datasets. As shown, interactions have clear peak (color coded in red) in the highly reproducible region (upper-right region).

The relative abundance of proteins in each fraction were measured based on label-free MS2 ion intensity-based approaches (Detailed in the method section). To decipher physical associations from the data, we reasoned physically interacting proteins would show similar patterns across the biochemical fractions. The employed bioinformatics approach for the inference of co-fractionation networks based on the observed patterns is summarized in Fig 1B. As described below, for stringent analysis of data, physically interacting protein pairs were predicted based on five criteria: 1) showing significant similarity in their fractionation patterns; 2) being robust to the addition of noise in their observed co-fractionation similarity; 3) showing shared-peak in at least one fraction; 4) being stably interacting with protein complexes as judge by GG-derived patterns; 5) reproducibility of co-fractionation when the same fractionation technique was used. Initially, we constructed four separate co-fractionation networks by application of the context likelihood relatedness (CLR) algorithm [34] on each of four datasets, separately. As described in the methods section, the CLR algorithm is an information theoretic-based approach that can predict the association of two random variables (proteins) based on their observed values (fractionation patterns). It predicts two proteins as interacting only if the similarity of their patterns is significantly higher than what expected based on the background (estimated Benjamini-corrected FDR of 5%). Low abundance proteins might not have reliable patterns in the dataset and therefore show random similarities to other proteins in their fractionation patterns. To confirm that observed similarity is not due to presence of noise in the dataset, co-fractionation similarities were recalculated after the generation of Poisson noise models from each dataset. In each network, we discarded those interactions that lost their significant similarity due to the addition of noise (Detailed in the method section). To further reduce the possibility of chance co-fractionation, we only kept those interactions for which both interacting proteins had a peak in at least one shared fraction. A protein was deemed to have a peak in a fraction if its ion intensity for that fraction was at least 80% of its second-highest intensity. We observed that filtering the interactions by this more stringent criterion led to an increased reproducibility rate of results and, most likely, the elimination of putative false positive interactions from the networks (Fig 3A). In GG fractionation experiments, early fractions are highly enriched for monomeric proteins in the cell (e.g., many enzymes) or those that are not stably involved in the complexes. Since interacting partners for these groups of proteins cannot be reliably identified by our approach, proteins that had a peak only in the top two fractions of GG experiments were discarded from the networks. Cytosolic-IEX and mitochondrial-IEX networks were significantly depleted for proteins of one another (p-value < 6E-35), most likely because of the enrichment procedures used for these two experiments. To check the technical reproducibility of the results, we focused on interactions occurring among proteins that were detected in both GG experiments. As illustrated in Fig 3B, we found that more than half of co-sedimented protein pairs (940 reproducible interactions) in one GG experiment were also co-sedimented in the other (FDR ≤0.05 in one experiment and p-value ≤0.05 in the other). Moreover, comparing the number of common interactions between whole cell-GG and mitochondrial-GG networks with random networks with the same structural characteristics revealed that these two networks are significantly enriched for reproducible interactions (S3 Fig). These results indicated that significantly co-sedimented protein pairs in the whole cell-GG experiment are typically co-sedimented in the mitochondrial-GG experiment and *vice versa*. To increase the accuracy, we removed the interactions among those proteins that were not consistently co-fractionated with each other in both experiments (i.e., despite the detection of proteins in both experiments, they co-fractionated in only one experiment). Overall, merging GG networks led to a network composed of 12,196 interactions among 1,417 proteins. The IEX experiments led to the identification of 1,708 interactions among 1,261 proteins. The smaller size of IEX network compared to those of GG is because former experiments had smaller number of fractions and, therefore, were less informative on protein complexes compared to GG experiments. We next assessed the agreements between IEX-derived and GG-derived networks. As shown in Fig 3C, comparison of cytosolic-IEX network with whole cell-GG network indicated that interactions have peak in the reproducible region (median correlation of 0.47 and 0.83 for cytosolic-IEX and whole cell-GG, respectively), but skewed toward the region with negative IEX correlations. Importantly, none of significantly co-eluted protein pairs in cytosolic-IEX experiment had correlations less than 0.47 in the whole cell-GG dataset. Comparison of mitochondrial-IEX network with mitochondrial-GG network led to a similar result (Fig 3D); i.e., interactions had peak in the reproducible region (median correlation of 0.53 and 0.73 in mitochondrial-IEX and mitochondrial-GG datasets, respectively). Moreover, comparison with random graphs demonstrated that the IEX-derived and GG-derived networks were significantly enriched for the reproducible interactions (S4 Fig). Due to separation of protein complexes in IEX and GG experiments based on different biochemical properties, the confounding complexes in one approach have lower chance to co-fractionate in the other approach as well. However, the observed discrepancies in co-fractionation patterns are not only because of confounding elements, but rather reflect the differences in the nature of the two fractionation experiments as well. As an illustration, we observed that members of translation initiation complex co-sedimented in the whole cell-GG network with the median correlation of 0.85. However, their median correlation in the cytosolic-IEX experiments reduced to 0.53. Likewise, comparison of the co-fractionation patterns among a set of proteins enriched for RNA-dependent interactions demonstrated their strong co-sedimentation in the GG experiments, but separation in the IEX experiment (S5 Fig). This discrepancy can be due to the use of salt gradient in the latter experiment and disruption of the less stable ionic interaction complexes. However, these effects were minimal on complexes that are known to form more stable complexes such as ribosome, proteasome, F0F1 ATPase, and core editosome (discussed more on the validation part). These results suggested that our GG fractionation experiments better preserved the less stable interactions (i.e. ionic-based interactions), but our IEX experiments favored more stable interactions. For the follow-up analysis, we merged the GG and IEX networks together. This global physical map, named TbCF net (*T*. *brucei* co-fractionation network), was constructed by distinguishing two types of interactions (S2 Table): 1) Those protein pairs that were reproducibly co-fractionated in both GG and IEX networks (FDR ≤0.05 in one experiment and p-value ≤0.05 in the other); and 2) Those protein pairs that were co-fractionated only in one experiment. The TbCF net connects 2,151 proteins with 13,865 interactions. The orthogonal reproducible part of TbCF network, termed TbCF_OR_ net, was composed of 2,601 (19% of total) interactions among 828 (38% of total) proteins.

### Consistency of the predicted network with previous findings

**Fig 4:**
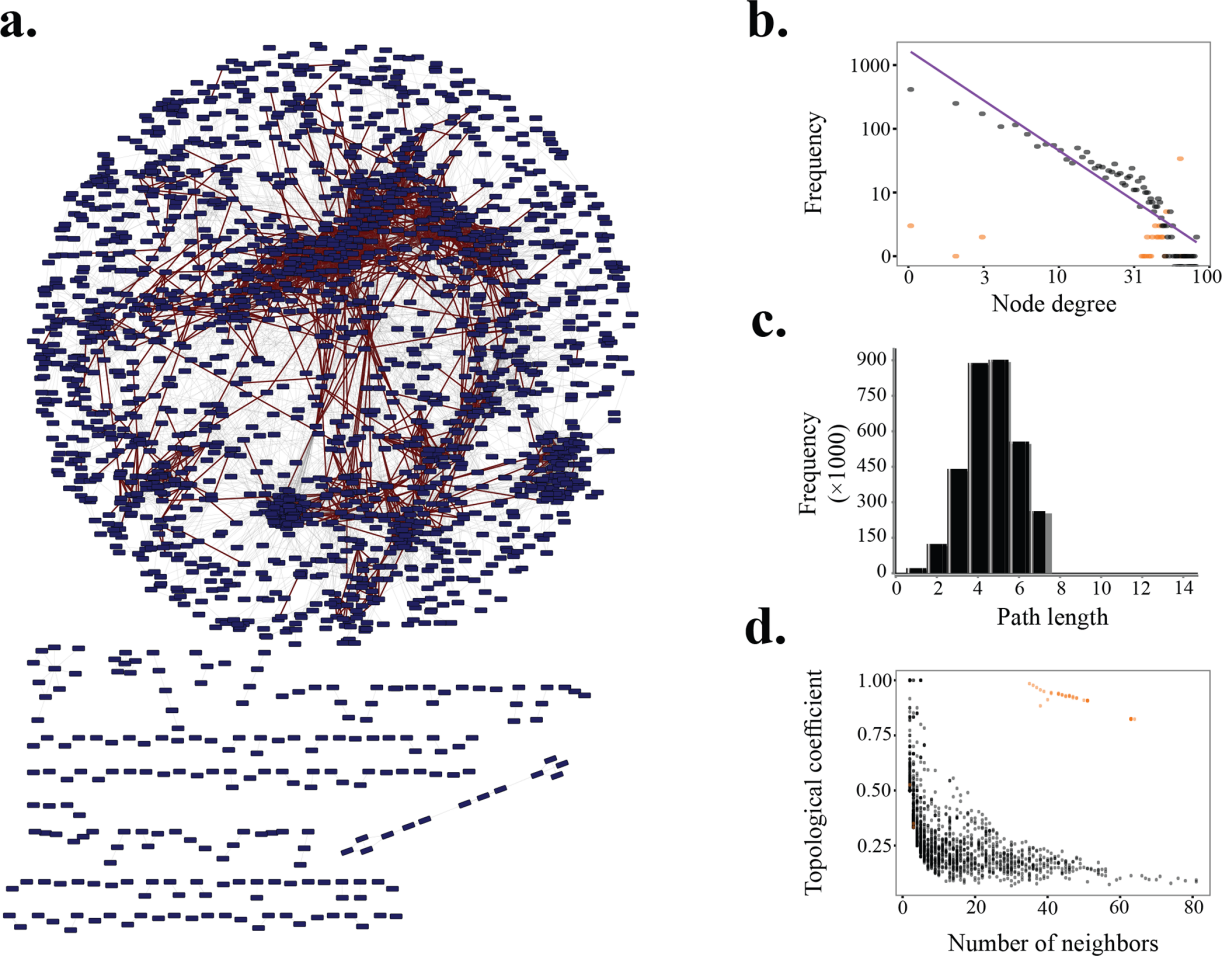
Topological characteristics of TbCF net. a) Representation of TbCF net. Red interactions correspond to those that were reproducibly identified in both GG and IEX fractionation experiments. b) Distribution of number of nodes as a function of number of node neighbors (i.e., node degree) in logarithmic scale. Interactions between ribosomal and non-ribosomal proteins were analyzed separately and were distinguished in the graph by yellow and black colors, respectively. c) Distribution of shortest path lengths in the TbCF net. In accordance with the small world property of biological networks, the path length is usually small with a unimodal peak at five. To avoid bias, ribosomal proteins were discarded for this analysis. d) Distribution of the topological coefficient of nodes as a function of node degree. Similar to hub degree analysis, interactions between ribosomal and non-ribosomal proteins were analyzed separately and were distinguished in the graph by yellow and black colors, respectively.

To test the validity of TbCF net, we first examined the topological properties of the constructed network (Fig 4A). In agreement with protein interaction networks of model organisms, TbCF net has scales-free architecture [53]; i.e., while most proteins interact with a small number of proteins, some of them (known as hubs) are highly linked to the other proteins (Fig 4B). Moreover, as shown in Fig 4C, each protein typically can be reached from every other protein by a small traverse in the network (i.e., small path length), as expected from a network with a small-world property [54]. Additionally, we observed that expect for ribosomal proteins which make a large, densely intra-connected module in the TbCF net, the topological coefficient also decreases with the number of neighbors (Fig 4D), reflecting that the number of common neighbors for hub proteins compared with the other proteins in the network is relatively low. This feature indicates that highly interacting proteins are not sporadically connected to each other.

**Fig 5:**
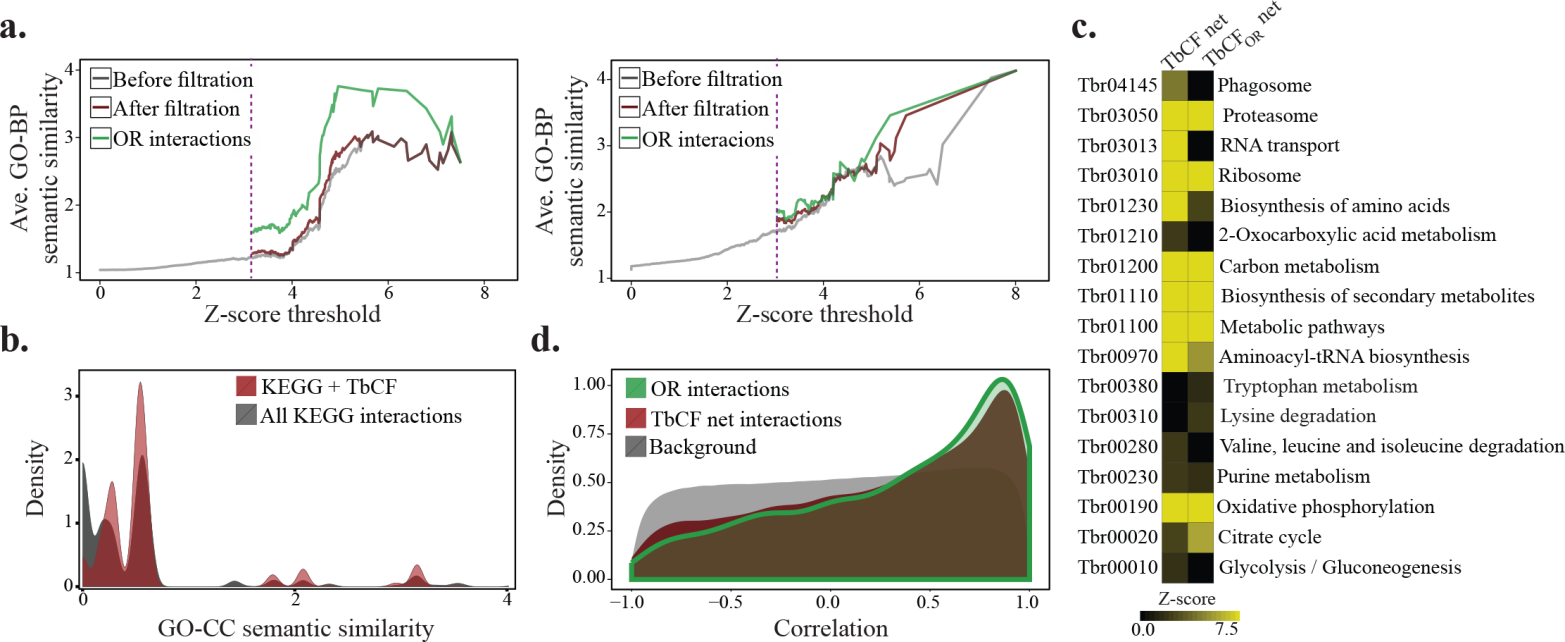
Biological validation of the constructed co-fractionation network. a) The average GO-BP semantic similarity was calculated across different z-score cut-off thresholds for whole cell-GG and mitochondrial-GG experiments, separately. The purple line highlights the co-sedimentation cut-off threshold corresponding to a false discovery rate of 0.05. To examine the applied filtration steps (elimination of noise sensitive, unshared-peak, early sedimenting, and non-reproducible interactions), we applied the same analysis to whole cell-GG and mitochondrial-GG networks before and after the filtration steps. As shown, the employed filtration steps have led to an increase in precision in both networks. However, the reproducible interactions constantly had higher similarity compared to the non-reproducible interactions. b) GO-CC semantic similarity distribution of all possible protein pairs with the same KEGG pathway was compared with the distribution of those KEGG interactions that were also supported by TbCF net. As illustrated, protein pairs in the latter group have significantly higher (p-value <3.5e-33, Wilcoxon-Mann-Whitney rank sum test) co-localization scores compared to the all functional interactions supported by KEGG. c) KEGG pathway modulation analysis of TbCF and TbCF_OR_ networks. The graph is pseudo-colored where the yellow color indicates significant connections (p-value <0.05) among proteins in the corresponding KEGG pathway. d) Distribution of observed co-expression similarities among all proteins detected in our study (background), protein pairs interacting in TbCF net, and those in TbCF_OR_ net. Gene expression data were extracted from [50]. A more comprehensive analysis is presented in S8 Fig. OR interactions: Orthogonally reproducible interactions; KEGG + TbCF: KEGG interactions supported by TbCF network.

We next assessed different biological features that can be expected from a protein interaction network. To this end, we first examined whether interacting proteins in the network tend to be involved in the same biological process. As shown in Fig 5A and S6 Fig, we observed that higher similarity scores of fractionation patterns consistently led to an increased probability that two proteins are involved in the same biological process, as judged by gene ontology-biological process (GO-BP) analysis. Importantly, this analysis suggested the higher precision of TbCF_OR_ net compared to the TbCF net. KEGG pathway [55] analysis also led to similar results, i.e., the chance of two proteins participating in the same KEGG pathway increases by having a higher similarity score (S7 Fig). Although proteins in the same KEGG pathway do not necessarily interact with each other as they can have functional rather than physical associations, protein complexes are involved in some KEGG pathways such as ribosome and proteasome. Importantly, we found that KEGG pathway interactions that were also supported by the TbCF net had significantly higher (p-value <3.5E-33, Wilcoxon-Mann-Whitney rank sum test) co-localization scores compared with the other KEGG interactions (Fig 5B), suggesting the physical nature of associations for the captured interactions. In a more stringent analysis, we next investigated whether or not proteins involved in a same KEGG pathway tend to form a significant module in the TbCF net. To this end, we used a score, termed modulation score [36], that assess the density of connections among a set of pre-specified proteins; e.g., proteins with a shared KEGG attribute (See methods for details). This analysis revealed that members of 15 KEGG pathways form densely connected modules (p-value<0.05) in the TbCF net (Fig 5C). Interestingly, we observed a significant modulation score for many pathways that are well-known to involve protein complexes (ribosomes, proteasome, RNA transport, oxidative phosphorylation, etc.). The same analysis on the TbCF_OR_ net indicated that this network is not biased towards a specific process and most KEGG pathways remain or even become significantly connected in the TbCF_OR_ net. However, as discussed earlier, TbCF_OR_ net were depleted for those pathways that involved less stable and salt sensitive interactions (Fig 5C). We also analyzed the TbCF net in terms of gene expression responses. In model organisms, it has been shown that transcriptional co-regulation of proteins plays a major role in the efficient control of the cell homeostasis in different environments [17]. It has also been shown in *T*. *brucei* that functionally-related proteins tend to be co-expressed with each other [56]. Indeed, gene expression analysis indicated that interacting proteins in the TbCF net show significantly high (p-value < 2.2E-16, Wilcoxon-Mann-Whitney rank sum test) co-expression trends in different life stages and also during the differentiation process (Fig 5D and S8 Fig). Consistent with GO-BP semantic similarity analysis, co-expression analysis also indicated a significantly higher (p-value < 2E-07, Wilcoxon-Mann-Whitney rank sum test) co-expression trend for interactions of TbCF_OR_ net compared to those of TbCF net, suggesting a higher precision of the former network.

### Extracting the high-confidence subset of TbCF net

**Fig 6:**
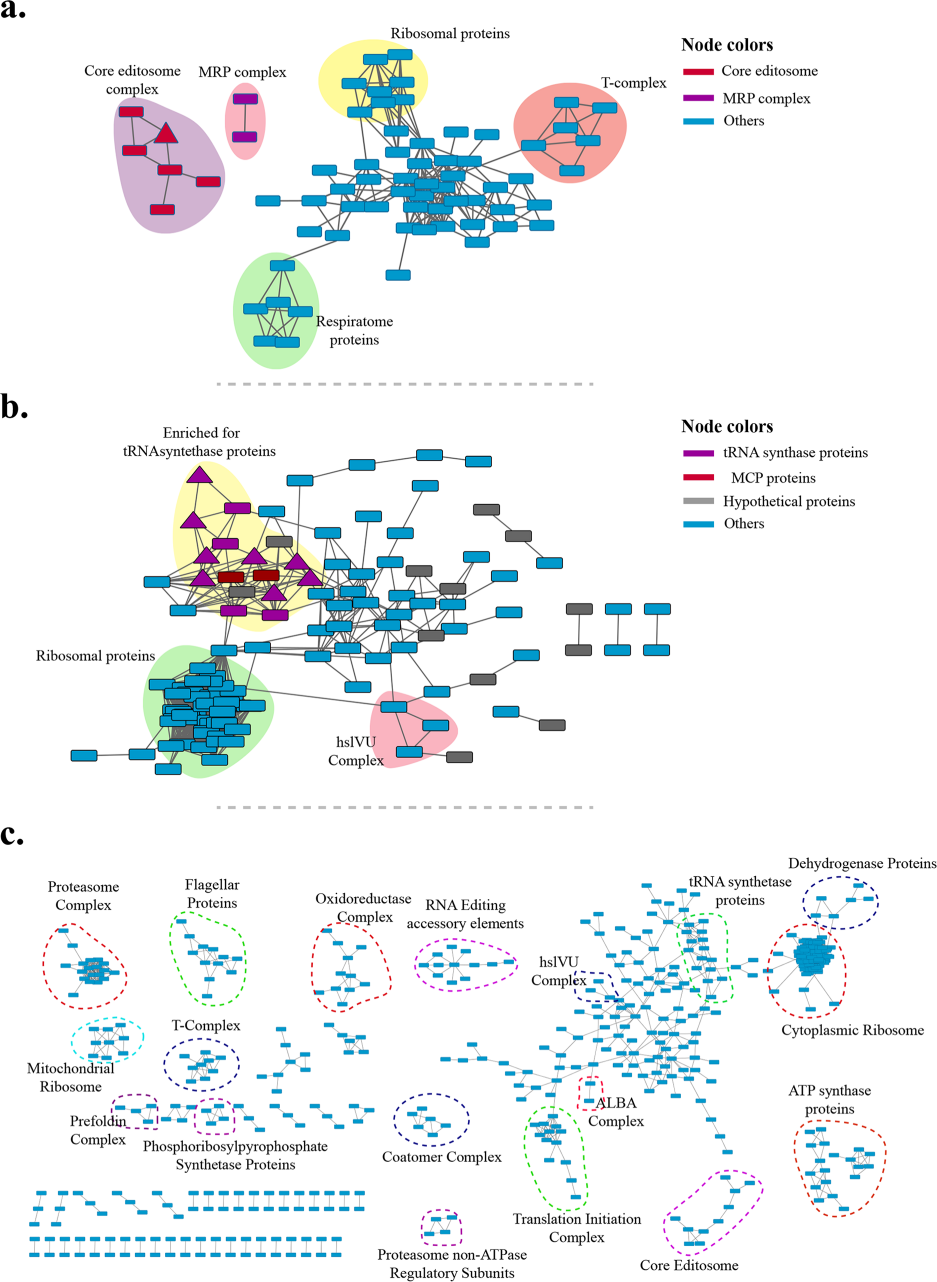
Integration of TbCF net with other highly contaminated resources. a) The sub-network of TbCF net that was restricted to 83 proteins co-purified with REL1 protein in the TEV-elution experiment. The REL1 protein is represented as a triangular node. b) The tRNA synthase sub-network, which was constructed by restricting TbCF net to 262 proteins that co-purified with tRNA synthase proteins in six independent pull-down experiments. The proteins identified in these pull-down experiments are represented as rectangular nodes. Interestingly, TbCF net suggested that some of the other tRNA synthase proteins, although not detected in these pull-down experiments, were significantly co-fractionated with the subunits of MARS complex. These additional tRNA synthase proteins are represented as triangular nodes. The list of *T*. *brucei* tRNA synthase proteins was extracted from [1]. c) Representation of the TbCF_STRING_ net which was constructed by considering the common sub-network between TbCF net and the STRING network. The associated protein names with these three graphs are included in S11 Fig and S1 File.

To estimate precision of TbCF net, we focused on twenty complexes that have been identified experimentally in *T*. *brucei*, composed of more than 200 proteins (S3 Table). This analysis demonstrated a precision of 34% in TbCF net with a recall of 80% on proteins and 17% on interactions, which is comparable to previous high-throughput studies of protein interactions based on biochemical fractionation [10, 13] (see methods for details). However, the estimated precision can be an underestimate as in many cases, subunits of the complexes had been partially identified because of application of stringent conditions. Importantly, consistent with the expected higher precision of TbCF_OR_ net as judged by GO-BP and co-expression analysis, the same analysis on TbCF_OR_ net with literature interactions suggested a precision of 59% (~2-fold increase in precision). As mentioned in the introduction, the integration of different data sources is highly recommended to reduce false positive results from an interactome. However, due to the lack of a global, unbiased protein interaction map for *T*. *brucei*, we were not able to utilize the state-of-art machine learning approaches to systematically integrate additional data sources. To experimentally examine the extent to which the precision of TbCF network can be further improved by other orthogonal information, we performed TAP-TAG TEV-elution of RNA editing ligase 1 (REL1) protein. We selected this protein because it is a subunit of a well-studied *T*. *brucei* complex, the core editosome. TEV-elution is a low stringency condition in which transient interactors along with many contaminants can co-purify with the tagged protein. TEV-elution of REL1 protein led to the co-purification of 83 proteins as putative interacting partners (S4 Table). We extracted the sub-network from TbCF net that was restricted to the co-purified proteins. Consistent with that, REL1 was directly connected to its known interacting partners, while well separated from other contaminant proteins in the network (Fig 6A). It should be noted that since only a small subset (i.e., five proteins) of previously known interacting partners of REL1 protein were co-purified in the pull down experiment, and the constructed sub-network was restricted to the identified proteins in that experiment, the other previously known interacting proteins that were also connected to REL1 in TbCF net are not represented in the sub-network, indicating the importance of queried proteins on obtaining a comprehensive sub-network. To further assess the TbCF net, we considered six separate pull down experiments of aminoacyl-tRNA synthetase (aaRS) proteins in different *T*. *brucei* life stages [57]. In these pull down experiments, 262 proteins were co-purified as candidate proteins involved in *T*. *brucei* tRNA-synthesis with a minimum overall mass spectral count of two. Following the same procedure as that used for REL1, the sub-network of these proteins suggested the existence of several distinct complexes among the proteins co-purified with aaRS proteins (Fig 6B). Interestingly, one of these complexes was highly enriched for proteins involved in tRNA-synthesis including the recently identified members of MARS complex [57] and two additional hypothetical proteins. Therefore, the TbCF net was able to successfully distinguish contaminant proteins from direct interactors in both cases. Integration of various TAP-TAG experiments with the TbCF net also suggested that the chance of retrieving a high false positive rate with a targeted search, i.e., a list of putatively interacting proteins, is very low. In our next attempt, we considered the functional protein interaction network of *T*. *brucei* deposited in the STRING database [58]. In the STRING database, most of available functional association data for *T*. *brucei* is inferred based on the indirect approaches (such as text mining and co-expression) rather than direct experimental evidence (S9 Fig). We extracted the STRING network using a medium confidence level threshold (the default threshold set by STRING) for the proteins present in the TbCF net. The retained STRING network was densely connected with 19,119 interactions among 1,402 proteins (S10 Fig). Next, we derived a secondary network composed of the interactions that were supported by both STRING and TbCF net, termed TbCF_STRING_ net. The TbCF_STRING_ net was composed of 2,413 interactions among 449 proteins (that included 13% of interactions and 32% of proteins in the primary STRING network, S10 Fig). To assess the validity of the network, the TbCF_STRING_ net was clustered using the clusterOne algorithm [59]. As shown in Fig 6C (and presented in more details in S1 File), many previously known protein complexes were recovered by this approach, indicating the high accuracy of the TbCF_STRING_ net. This result indicated that integration of our co-fractionation network with other independent sources (like AP and STRING in this case) leads to the elimination of false positive interactions from both sources.

Based on the findings in the previous step, we partitioned the interactions in the TbCF net into two parts; those with high confidence, TbCF_HC_, and those with no external evidence, TbCF_NE_. As schematically shown in Fig 1A, the high confidence group was composed of interactions that were supported by at least one of the orthogonal resources including KEGG pathways, the STRING database, interlog-mapping, extensive literature searches, and orthogonal reproducibility (see methods for details). As discussed earlier, interaction data in each of these orthogonal resources (e.g. KEGG, STRING, AP, etc.) suffers from false positives and also, for some sources, does not imply physical interaction among protein pairs. However, we expected to observe elimination of the false positive interactions by integration of these data with our fractionation-derived network. TbCF_HC_ network was composed of 4,726 interactions among 866 proteins (S2 Table), encompassing 34% of the total interactions in TbCF net (Table 1). Analysis of TbCF_HC_ net indicated its improvement over TbCF net in terms of GO-BP (S12 Fig; p-value <2E-08, Wilcoxon-Mann-Whitney rank sum test), GO-CC (S12 Fig; p-value <6E-07, Wilcoxon-Mann-Whitney rank sum test), and co-expression (S12 Fig; p-value <5E-117, Wilcoxon-Mann-Whitney rank sum test). As judged by the analysis of the same twenty protein complexes discussed above, the estimated precision for TbCF_HC_ net was 80% and 68% without and with the exclusion of literature-derived data from the network, respectively. The estimated precisions for both TbCF_OR_ and TbCF_HC_ networks suggest that integration of TbCF net with other orthogonal resources leads to the overall false discovery rate of less than 40%.

**Table 1:** Contribution of the orthogonal resources in verification of TbCF net.

Resource	No. of verified interactions
KEGG	2608 (19% of total interaction)
STRING	2413 (17% of total interaction)
interlog-mapping	318 (2% of total interaction)
Literature	1421 (10% of total interaction)
Orthogonal reproducibility	455 (3% of total interactions)
**Total**	**4726 (34% of total interaction)**

**Fig 7:**
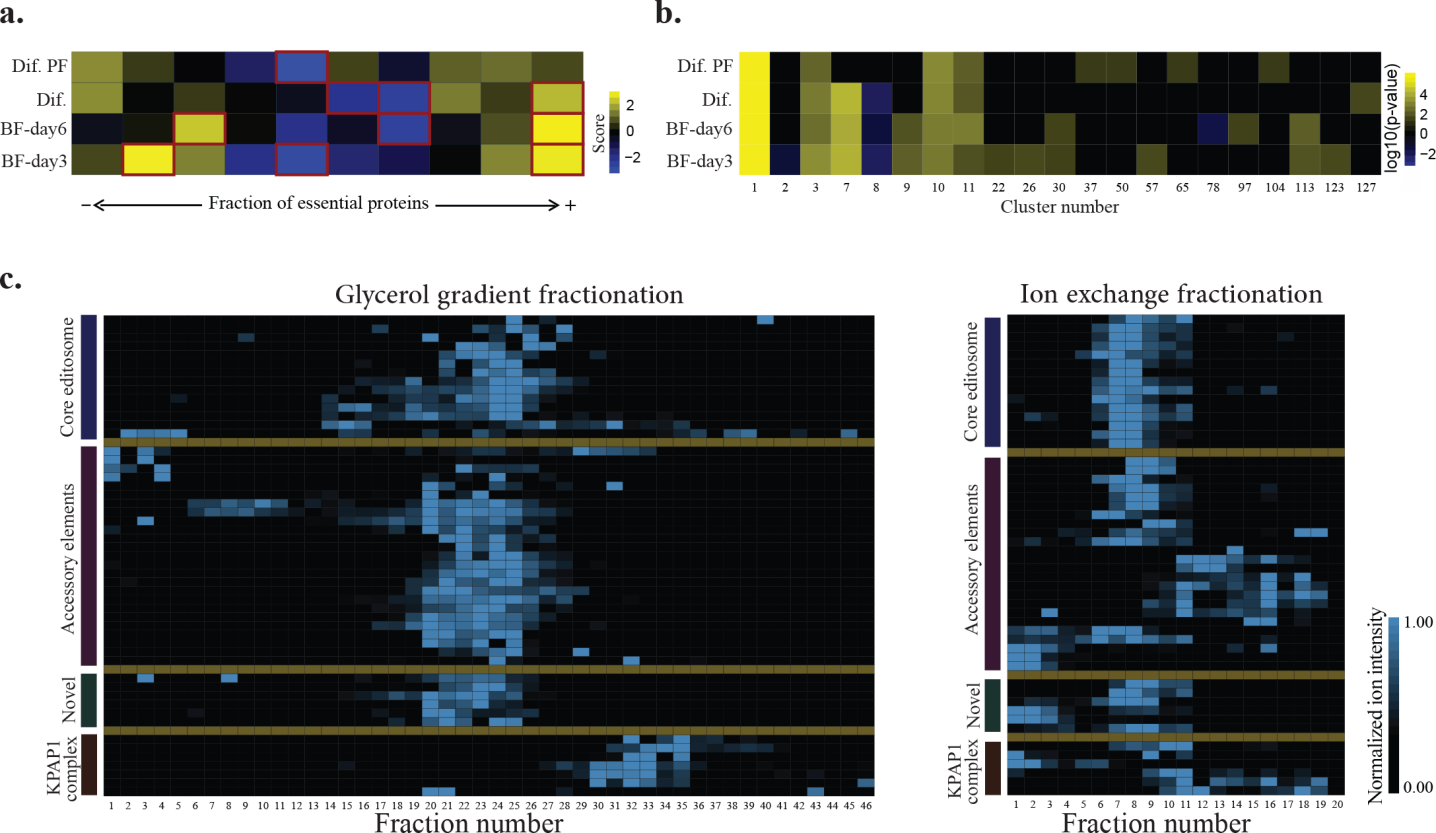
Evaluation of predicted complexes based on TbCF_HC_ net. a) The 128 predicted complexes were classified to ten uniformly spaced bins based on the fraction of essential proteins present in each complex. The number of complexes present in each bin was compared to the expected background distribution using MATLAB kernel smoothing function. The expected background distribution was estimated by applying the same clustering analysis (i.e., ClusterOne algorithm) to 10,000 random networks from TbCF net, generated by shuffling the protein labels, while preserving structural properties. Figure represents the calculated kernel score for each bin, with blue and yellow colors indicating under-representation and over-representation, respectively. Significantly enriched or depleted bins are shown by red borders. As shown, we observe over-representation of TbCF_HC_ net-derived complexes with enriched or depleted fraction of essential genes (corresponding to right and left bins, respectively). However, TbCF_HC_ net-derived complexes with random distribution of essential genes (corresponding to the bins in the middle) were under-represented. The list of essential genes in 4 different life stages of *T*. *brucei* was extracted from [60]. (Dif. PF: differentiated procyclic cells; Dif.: Differentiating cells from bloodstream to procyclic; BF-day6: Representing bloodstream stumpy form; BF-day3: Representing bloodstream short slender form). b) The 128 predicted complexes were examined for possible enrichment of essential proteins in different life stages and during the differentiation process from bloodstream to the procyclic form (see above for abbreviations). The enrichment analysis was performed using Fisher’s exact test. At the p-value cut-off threshold of 0.05, the yellow color indicates a significant over-expression and the blue color represents a significant under-representation of essential proteins in a complex. It should be noted that most of predicted complexes had small sizes (median of four subunits per complex). Therefore, the statistical test did not have enough power to detect significant over-/under- representations of essential genes in these small complexes. Correspondence of previously known protein complexes to the reported complexes in this figure: cytoplasmic ribosomal complex (Complex 1), proteasome (Complex 3), and T-complex (Complexes 10 and 11), intraflagellar proteins (Complex 7), mitochondrial ribosome (Complex 2 and 8). c) GG and IEX fractionation patterns for the proteins known/predicted to be involved in the RNA editing and KPAP1 complex. Proteins are categorized in four groups of core editosome, accessory elements, novel proteins, and KPAP1 complex. The associated protein names are included in S14 Fig.

Clustering TbCF_HC_ net using ClusterOne algorithm [59] led to the prediction of 128 protein complexes among 716 proteins (S5 Table). The predicted complexes varied in size between three and 70 with the median size of four proteins per complex that is similar to reports from other organisms [10]. Many of the predicted complexes were significantly enriched for subunits of previously known *T*. *brucei* complexes. For example, as illustrated in S13 Fig, we were able to successfully identify complexes related to cytoplasmic and mitochondrial ribosomes, proteasome, T-Complex, intraflagellar transport, translation initiation, and mitochondrial RNA editing. To further assess the quality of predicted complexes, we associated them with the available large-scale RNAi screening data for *T*. *brucei* [60]. Because each protein complex act as a functional unit in the cell, it is expected that the essential proteins to be over- or under-represented in a protein complex, depending on the function of the complex [61]. Indeed, enrichment analysis of predicted complexes indicated over representation of complexes with enriched or depleted fraction of essential proteins, while complexes with random distribution of essential proteins were under represented (Fig 7A). The predicted essential complexes recapitulated the previous findings for *T*. *brucei*. As an illustration, the results indicated that the cytoplasmic ribosomal complex (Complex 1), proteasome (Complex 3), and T-complex (Complexes 10 and 11) are essential in all life stages of the parasite (Fig 7B). Conversely, the complex 7 which is highly enriched for intraflagellar proteins (Fig 7B), was not essential in the procyclic life stage of *T*. *brucei* [62]. Additionally, complexes related to the mitochondrial ribosome (Complex 2 and 8) were depleted for the essential proteins in the bloodstream life stages (Fig 7B).

### Validation of TbCF_HC_ net

Clustering of TbCF_HC_ net also led to the prediction of complex membership for 188 protein groups currently annotated as hypothetical, 350 protein groups with the annotated name as putative, and 635 protein groups lacked experimental GO-BP annotation (evidence codes of EXP, IDA, IPI, IMP, IGI, and IEP according to TriTrypDB v5). To experimentally verify the quality of predictions, we focused on the overlapping complexes that were highly enriched for proteins involved in the Kinetoplastid RNA editing process.

Mitochondrial gene regulation is a highly interesting, yet not fully understood, process in *T*. *brucei*. In this process, mitochondrial genes are transcribed as polycistronic units. After cleavage, mitochondrial transcripts (mtRNAs) become stabilized by the addition of short tails at their 3' ends [63]. Intriguingly, most of the produced mtRNAs originally do not possess correct open reading frames (ORFs) and require to be edited before the translation. In the editing process, small RNAs, known as guide RNAs (gRNAs), dictate the insertion, and less frequently deletion, of a defined number of uridine nucleotides at pre-specified positions in mtRNAs [64]. The edited mtRNAs become marked for the translation by the addition of long A/U tails [65]. This highly complicated and intertwined process provides the parasite multiple post-transcriptional regulatory layers over mtRNAs. Mitochondrial post-transcriptional gene regulation is essential for the survival of the parasite in both bloodstream and procyclic life stages, although the precise role of this process is not well understood in the bloodstream form [66, 67]. Experimental evidence confirms differential regulation of some mtRNAs in at least editing step during the parasite’s life cycle [68, 69]. For example, while the Cytochrome b (Cyb; a subunit of complex III) and cytochrome oxidase subunit II (COII) transcripts are preferentially edited in procyclic form, some subunits of complex I such as NADH-ubiquinone oxidoreductase subunit 8 (ND8) and NADH-ubiquinone oxidoreductase subunit 7 (ND7) are mostly edited in the bloodstream stage. This developmental regulation in RNA editing is coordinated with the activities of the trypanosome mitochondria during its life cycle to allow the adaptation and survival of the organism in changing environmental conditions [70]. Consistent with the developmental regulation of the RNA editing process, comparative sedimentation analysis of the RNA editing machinery demonstrated that the complexes associated with the RNA editing machinery of bloodstream and procyclic forms are not identical [71].

The TbCF_HC_ net suggested the physical association of 49 protein groups (50 proteins) with the RNA editing machinery of *T*. *brucei* in the procyclic life stage (S6 Table). Many of these proteins are well-known to be involved in the RNA editing process. For example, among the 50 predicted proteins, 17 are known to be involved in the core editosome complex [72], while 21 function in the MRB1 complex [73]. For detailed analysis of their interactions, we distinguished predicted interactions originated from IEX experiments to those of GG experiment. This analysis indicated that members of core editosome and some of the accessory elements were reproducibly co-fractionated in both approaches (S15 Fig). However, about 70% of significantly co-sedimenting protein pairs in the GG experiments were not significantly co-eluted in the IEX experiments (S15 Fig). Visual inspection of the fractionation patterns of these 50 proteins confirmed that they mainly co-sedimented together in mitochondrial-GG experiment, but dissociated into different protein clusters in the mitochondrial-IEX experiment (Fig 7C). It should be pointed out that many proteins that are functionally associated with the RNA editing machinery, mediate their functions through binding to the RNA and GG and IEX mitochondrial fractionation experiments were not RNase treated. However, technical differences between the IEX and GG experiments provided a high resolution picture of the distinct complexes involved in the mitochondrial post-transcriptional regulation of *T*. *brucei*. For example, while members of core editosome were reproducibly co-fractionated in both approaches, comparison of IEX and GG data suggested existence of at least three distinct groups of proteins among the accessory elements. These three groups were co-sedimented with each other and with core editosome in the mitochondrial-GG experiment. However, they failed to co-fractionate in the mitochondrial-IEX experiment possibly due to the increased salt concentration, suggesting RNA-dependent or less stable nature of interactions between these groups. Consistent with previous reports [73], observed fractionation patterns supported a direct interaction between Tb927.8.8170 and Tb927.11.16860, while suggested RNA-dependent interaction of Tb927.2.6070 with MRB core proteins (i.e., Tb927.5.3010, Tb927.10.11870, and Tb927.10.10130). However, comparison with previous findings on the interactome of proteins related to the RNA editing machinery suggested that the lack of co-elution in the IEX data could be also because of less stable direct protein-protein interactions. For example, although MRB8170 (Tb927.8.8170) and TbRGG2 (Tb927.10.10830) did not co-eluted in the IEX experiment, they were reported to directly interact in Y2H assays [73, 74], or AP-based studies [75]. Importantly, a previous study has demonstrated the TEV co-elution of the two proteins in a RNA-enhanced manner [76]. Hence, the lack of co-elution in the IEX fractionation experiment for MRB8170 and TbRGG2 proteins is likely due to less stable interaction of the two proteins. Moreover, fractionation data indicated that members of KPAP1 polyadenylation complex reproducibly fractionated differently from those of core editosome, and suggested their association with mitochondrial ribosome, which is consistent with their functional role that couple the mitochondrial editing with the translation process [65]. Also, the fractionation patterns successfully captured interactions of KPAP1 protein (Tb927.11.7960) with both editing and ribosomal complexes (Fig 7C), recapitulating the results obtained by mass spectrometric and immunochemical experiments [65]. The TbCF_HC_ net also suggested that six new proteins (Tb927.1.3010, Tb927.10.7910, Tb927.1.1730, Tb927.10.5830, Tb927.6.1200, and Tb927.10.1730) play a role in the mitochondrial post-transcriptional gene regulation. Fractionation patterns of GG experiment for these candidates indicated their co-sedimentation with core editosome and accessory elements, but separation from subunits of KPAP1 polyadenylation complex. Importantly, IEX fractionation patterns suggested that two of these candidates (Tb927.6.1200 and Tb927.10.1730) strikingly co-fractionated with members of the core-editosome and some accessory elements, but the co-elution of the other four proteins were, in varying degrees, sensitive to the presence of salt with Tb927.10.7910 being the most salt sensitive one.

**Fig 8:**
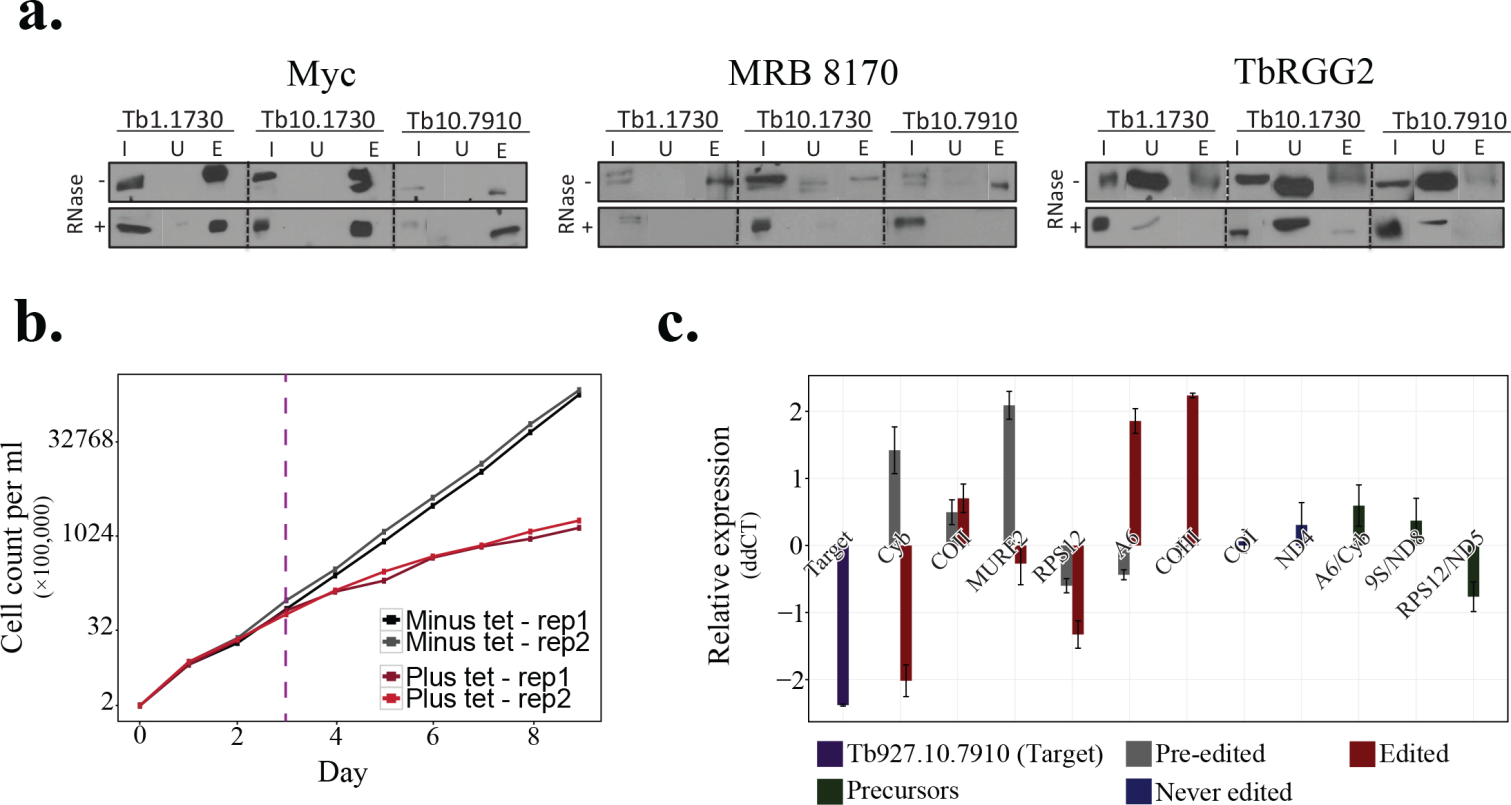
Experimental validation of the candidate proteins. a) Tb927.1.1730 (Tb1.1730), Tb927.10.1730 (Tb10.1730) and Tb927.10.7910 (Tb10.7910) proteins possess interactions with TbRGG2 sub complex.Immunoprecipitation of cmyc-Tb927.1.1730, cmyc-Tb927.10.1730, and cmyc-Tb927.10.7910 from mitochondrial extracts either RNase inhibited (-RNase) or RNase treated (+RNase). Proteins from input (I), unbound (U), and eluate (E) were electrophoresed on 10% SDS-polyacrylamide gel and the blot was probed with specific antibodies against myc (to detect cmyc tag), MRB8170 and TbRGG2. b) Growth curve for Tb927.10.7910 RNAi-knock down experiment. The dashed purple line indicates the selected day (day-3) for collecting RNA sample to examine the knock down effect on mitochondrial transcripts. c) The RNA-editing machinery works on pre-edited mRNA substrates to make edited mRNA; therefore, interfering with this machinery is expected to lead to accumulation of pre-edited and down-regulation of edited mRNAs. We observed this phenotype for the Cyb transcript in knock-down experiment of Tb927.10.7910. The fact that some other edited and pre-edited mtRNAs are also affected can be suggesting that the protein plays multiple functions in the mitochondria gene regulation network of *T*. *brucei*. The knock down experiment was performed with two independent biological replicates and three technical replicates (six replicates in total).

Examining the localization pattern for three of our candidate proteins (Tb927.10.7910, Tb927.10.1730, and Tb927.1.1730) clearly confirmed their exclusive presence in the mitochondria of the parasite (S16 Fig). We also performed immunoprecipitation experiments on tagged versions of these three proteins. These experiments verified their interactions with TbRGG2 and MRB8170 proteins. Consistent with the observed IEX fractionation patterns, we found that the interaction of all three candidates with MRB8170 was abolished following the RNase treatment (Fig 8A). Likewise, we found that only Tb927.10.1730 remains still bound to TbRGG2 after RNase treatment (Fig 8A). Consistent with this work, another study reported four out of our six candidates (including Tb927.1.3010, Tb927.1.1730, Tb927.6.1200, Tb927.10.1730) form a novel complex involved in the post-transcriptional regulation of mtRNAs, termed polyadenylation mediator complex, confirming the predictions on these four proteins [77]. To further assess the role of the candidate proteins in the RNA editing process, we performed tetracycline (Tet)-inducible RNA interference (RNAi) knockdown of Tb927.10.7910 in the procyclic form of *T*. *brucei*. RNAi induction for this gene led to growth-defect phenotype, reflecting its essential role in normal growth of the parasite in the procyclic life stage (Fig 8B). Follow up quantitative RT-PCR verified the knockdown of the candidate transcript compared with the control, uninduced cells (Fig 8C). We next quantified the relative changes in mitochondrial-encoded pre-edited, edited, and never-edited transcripts in the RNAi-knock down background. We also considered three precursor RNAs to examine whether this protein play a role in the precursor RNA processing. This experiment indicated that Tb927.10.7910 affect the RNA editing process as judged by the accumulation or reduction of pre-edited or edited transcripts for different target RNAs (Fig 8C). Interestingly, our results suggested that Tb927.10.7910 affect the editing process of the Cyb transcript (i.e. upregulation of pre-edited mRNA and down-regulation of the edited mRNA). The knock down of Tb927.10.7910 also led to the down regulation of RPS12 edited transcript, and also accumulation of MURF2 pre-edited as well as COIII and A6 edited mtRNAs, suggesting the multiple functionality of Tb927.10.7910 in the mitochondrial post transcriptional regulatory network of *T*. *brucei*. Consistently, a previous study has suggested the essentiality of Tb927.10.7910 protein in the bloodstream life stage of the parasite [60].

### Conclusions

We have presented a systematic study of protein complexes in *T*. *brucei* using two complementary biochemical fractionation approaches. Our results led to the assignment of many previously uncharacterized proteins to complexes. The quality of predictions was verified by independent follow up experiments on newly characterized proteins associated with RNA editing machinery. Interestingly, we found that at least five out of six predictions of TbCF_HC_ net are truly associated with the RNA editing machinery, and also that one of them preferentially affect the editing process of Cyb transcript, a developmentally regulated mitochondrial mRNA. Further experiments are required to clarify the roles of these proteins in the mitochondrial gene regulatory network of *T*. *brucei*.

Despite the employment of stringent filtration criteria, our preliminary network (TbCF net) contains false positive interactions (estimated precision of 34%) that arose from limitations of the employed fractionation approaches. However, integration of results from two different fractionation approaches led to significant boosting of the precision (~2-fold), reflecting the importance of data integration for accurate predictions. Due to the lack of an unbiased, high-confidence, and large-enough protein complex map for *T*. *brucei*, we were not able to apply sophisticated machine-learning approaches to increase the precision of TbCF net by incorporating other data sources such as transcriptome. The KEGG interactions imply functional rather than physical associations. The interactions deposited in the STRING database were mostly inferred based on the indirect evidences that support functional associations. The literature-derived network was highly biased towards specific protein complexes and also contaminated with non-specific interactors. According to the primary literature-derived network, for example, there were ~360 co-purified protein groups with known members of RNA editing machinery. Of these, 242 protein groups were detected in our experiments. However, the TbCF net predicted strikingly similar co-fractionation pattern for only 49 of these protein groups, reflecting the high-contamination rate of literature-derived network. By stringent filtration of data, we focused on the high confidence sub-network of TbCF net (TbCF_HC_ net), composed of interactions whose existences were supported by at least one other independent resource. The TbCF_HC_ net refines and extracts new information from previous data and computational predictions on the interactome of *T*. *brucei*. However, this additional step can lead to the loss of information on proteins for which these types of evidences were not available. Therefore, we can expect that the number of high-confidence interactions will increase with the availability of more experimental data on *T*. *brucei* protein-protein interactions. Consistent with this, we have found that integration of results from other experiments (e.g. AP or immunoprecipitation) with TbCF net (which is a mixture of high- and low-confidence interactions) leads to the elimination of false positive results from both sources. Importantly, our analysis suggested that integration of TbCF net with other orthogonal resources leads to the overall false discovery rate of less than 40%. This finding has a great impact since most protein interactions in *T*. *brucei* are inferred by applying stringent conditions at the expense of an increased false negative rate, i.e., losing transient interactions. Our results suggest that this criterion can be relaxed by considering TbCF net as an orthogonal validation resource.

Mass spectrometry-based experiments are known to have limitations in the detection of low abundance proteins (including many regulator proteins), as they become masked in the sample by more abundant proteins. This issue was partially addressed in our approach by fractionating cell-extracts before mass spectrometry. Although comparable with previous SILAC experiments, our experiments cover 42% of proteins associated with expressed mRNAs in the procyclic stage [45]. Despite the fact that some of these transcripts can be translationally silent, the fraction of identified proteins is still potentially low. Our fractionation experiments demonstrated that enrichment for specific cellular compartments offers a viable solution to this issue. Hence, a greater depth could be obtained by performing similar experiments for other subcellular compartments of the cell such as the nucleus or mRNA enriched extracts. Moreover, our analysis clearly indicated an under-representation of membrane proteins in the mass spectrometry data that stems from the employed experimental procedure. Recovery of this class of proteins could likely be increased by use of detergents that can solubilize various membrane associated complexes and protein sub-domains.

The results of this work are deposited in a prototype version of a database available at: www.trypsNetDB.org.

## Supporting Information

S1 Fig Biological properties of the identified proteins in this study, related to the Fig 2. Comparison of number of transmembrane domains (**a**) and protein length (**b**) distributions for proteins identified in this study with those that are expected to be present in procyclic stage [45] and total predicted proteins in *T*. *brucei* (TriTrypDB v5).
(PDF)

S2 Fig Transcriptome characteristics of the identified proteins in different life stages and during the differentiation. Heatmaps represent expression patterns of the identified proteins in different life stages (**a**, extracted from *Jensen et al*. [50]) and during the differentiation process from the bloodstream to the procyclic form (**b**, extracted from *Kabani et al*. [52]) and (**c**, extracted from *Queiroz et al*. [51]). For each study, the expression data of each gene was normalized to have mean zero and standard deviation equal to one. The yellow color represents up-regulation and blue indicates the down-regulation. **d)** Plot represents average expression of transcripts against their standard deviations in five different life stages of *T*. *brucei* [50]. As shown, proteins identified in this proteomic-based study are biased towards more abundant transcripts in the cell.
(PDF)

S3 Fig Whole cell-GG and mitochondrial-GG networks are highly enriched for common interactions as judged by random graphs, related to Fig 3. One hundred different random datasets were generated by shuffling protein labels from each of the whole cell-GG and mitochondrial-GG datasets. Applying the same analysis pipeline as TbCF net, the distribution corresponding to the number of reproducible interactions (FDR ≤0.05 in one dataset and p-value ≤0.05 in the other) between each possible combination of random datasets (10,000 combinations in total) were observed. As illustrated, the expected number of reproducible interactions by chance is 137. However, the whole cell-GG and mitochondrial-GG networks share 940 reproducible interactions (the red arrow) with each other.
(PDF)

S4 Fig GG and IEX networks are highly enriched for common interactions as judged by random graphs, related to Fig 3. The four fractionation datasets were categorized based the employed fractionation approach to the IEX and GG groups. Within each group gene labels were randomized, while preserving the linkages between the datasets inside the group; e.g., if the gene label for geneA was shuffled to the gene99 in one dataset, the same gene was also called gene99 in the other dataset present in that particular group. This process repeated one hundred times for each group, generating one hundred random groups for each of IEX and GG groups. Networks were generated for each combination of groups applying the same criteria as those applied to construct TbCF net. Next, the distribution corresponding to the number of reproducible interactions (FDR ≤0.05 in one group and p-value ≤0.05 in the other) among each possible combination of random groups (10,000 combinations in total) were observed. As illustrated, the expected number of reproducible interactions by chance is 587. However, the GG-derived and IEX-derived networks share 2601 reproducible interactions (the red arrow) with each other.
(PDF)

S5 Fig Protein pairs with enriched RNA dependent interactions are over-represented among the non-reproducible interactions between IEX and GG networks, related to Fig 3. Interactions in each of the mitochondrial-GG and mitochondrial-IEX networks were categorized as either those occurring among proteins known to be associated with the RNA editing machinery or others. Yellow region demonstrates the area that is over-represented (p-value ≤0.05) for interactions among the proteins associated with the RNA editing machinery and blue demonstrates the regions with under-representation (p-value ≤0.05) of those interactions. Enrichment at each point on the graph was calculated using a two-tailed hypergeometric test by focusing on the closest 38 interactions to that point.
(PDF)

S6 Fig Biological validation of the constructed co-fractionation network, related to Fig 5. The average GO-BP semantic similarity was calculated across different z-score cut-off thresholds for the cytosolic-IEX and mitochondrial-IEX experiments, separately. The purple line highlights the co-elution cut-off threshold corresponding to a false discovery rate of 0.05. To examine the applied filtration steps (elimination of noise sensitive, unshared-peak, early sedimenting, and non-reproducible interactions), we applied the same analysis to the cytosolic-IEX network before and after the filtration steps, but not the mitochondrial-IEX network because of its small size. As shown, the employed filtration steps have led to an increase in precision. However, the reproducible interactions constantly had higher similarity compared to the non-reproducible interactions.
(PDF)

S7 Fig Biological validation of the constructed co-fractionation network, related to Fig 5. The percentage of protein pairs with a shared KEGG attribute was calculated across different zscore cut-off thresholds for all four fractionation datasets.
(PDF)

S8 Fig Interacting protein pairs in TbCF net are significantly co-expressed in most conditions, related to Fig 5. Pearson correlation coefficient was calculated between each of that interacting protein pairs in TbCF net (the red curves) and all possible pairs of the proteins identified in this study, as a control (the gray curves). Data from three datasets were used for this analysis [50–52]. As shown, the reproducible interactions (i.e., those co-fractionating in both fractionation approaches) constantly had higher similarity in terms of co-expression compared to the non-reproducible interactions.
(PDF)

S9 Fig Contribution of different inference methods on prediction of *T*. *brucei* interactome in STRING database. All interacting protein pairs related to *T*. *brucei* were downloaded from STRING v10 (5), and the average score for each inference method were calculated accordingly.
(PDF)

S10 Fig Integration of TbCF net with the STRING-derived network. **a)** Structure of extracted STRING network with the medium evidence score for proteins present in TbCF net. b) Structure of TbCFSTRING network that was generated by considering interactions that are present in both TbCF net and STRING-derived network. As illustrated, the integration has led to the generation of a more modular network.
(PDF)

S11 Fig A detailed view on the integration result of TbCF net with other available resources, related to Fig 6. (PDF)

S12 Fig Comparing the biological characteristics of TbCFHC net with those of TbCF net. Comparison of GO-BP, GO-CC, and Co-expression distributions indicates TbCF_HC_ is significantly improved over TbCF net. For co-expression analysis, data from [50] were used.
(PDF)

S13 Fig Graphical representation of TbCFHC net. As illustrated, clustering of TbCFHC net led to the recovery of many of previously identified complexes in *T*. *brucei*. Clustering also predicted some new complexes and assigned new members to the previously characterized complexes.
(PDF)

S14 Fig A detailed view on the cofractionation patterns proteins associated with *T*. *brucei* RNA editing machinery, related to Fig 7C. As illustrated, GG sedimentation patterns demonstrate the involvement of KPAP1 (in green color) protein with RNA-editing machinery, KPAP1 complex, and ribosomal proteins. Selected proteins for experimental validation are represented in red color.
(PDF)

S15 Fig The sub-network of TbCFHC net, related to complexes enriched for proteins involved in the RNA editing process. Clustering of TbCF_HC_ net predicted the involvement of 50 proteins in the RNA-editing machinery. This figure illustrates the interactions that were inferred based on the Mitochondrial-GG and Mitochondrial-IEX experiments. The edge color represents the source of experiment which interaction was inferred from.
(PDF)

S16 Fig Subcellular localization of three candidate proteins. Mitochondrial localization for C-terminal 2×myc–tagged Tb927.1.1730, Tb927.10.1730, and Tb927.10.7910 proteins. Anti-myc antibody was used to detect tagged cells in procyclic life stage. Mitochondrial localization was observed for all three genes after 48 hours of induction by Tetracyclin. Mitotracker was used to stain mitochondria and DAPI to detect kinetoplasts and nuclei. FITC (fluorescence isothiocyanide) was used to dye the tagged proteins. DIA (dialkyl aminos tyryl) was used to stain the parasite.
(PDF)

S1 Table Oligonucleotides used in this study. (DOCX)

S2 Table Co-fractionation networks introduced in this study. (XLSX)

S3 Table List of twenty complexes that were used to estimate the precision and recall of networks. (XLSX)

S4 Table TEV-elusion pull down of REL1 protein. (XLSX)

S5 Table Clustering results of TbGG-HC net. (XLSX)

S6 Table List of 50 proteins predicted to be associated with the RNA editing machinery. (XLSX)

S1 File A Cytoscape file containing the detailed information on the network represented in Fig 6C. (ZIP)
