# Supplementary figures and images for: A Protein Complex Map of Trypanosoma brucei
Source: PLoS Negl Trop Dis. 2016 Mar 18;10(3):e0004533. doi: 10.1371/journal.pntd.0004533 (PMC4798371; doi:10.1371/journal.pntd.0004533)

**a.**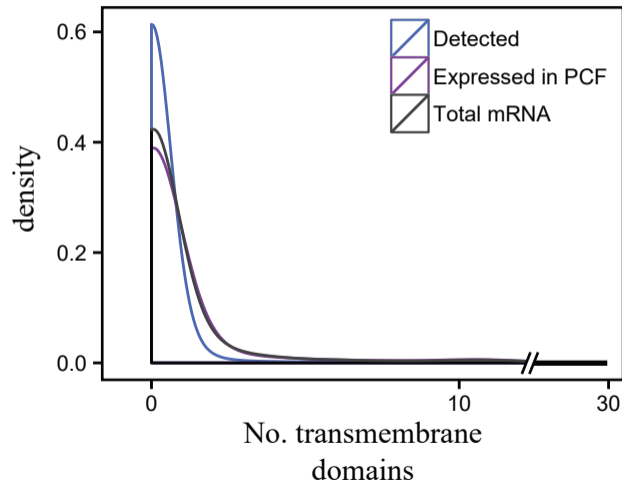**b.**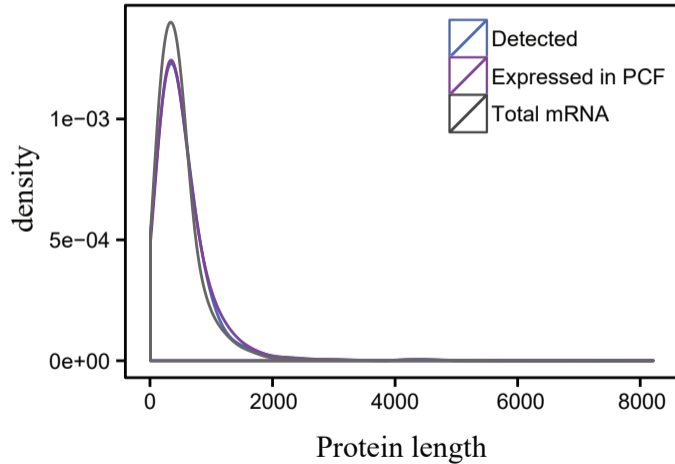

Supplement: S1 Fig — Comparison of number of transmembrane domains (a) and protein length (b) distributions for proteins identified in this study with those that are expected to be present in procyclic stage [45] and total predicted proteins in T. brucei (TriTrypDB v5). (PDF) [file pntd.0004533.s001.pdf]

**a.**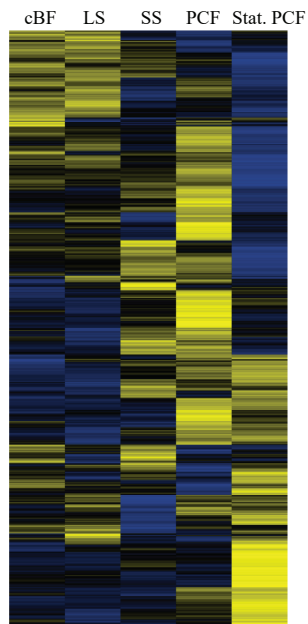**b.**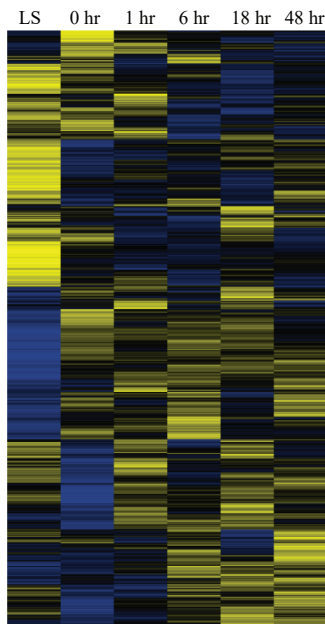**c.**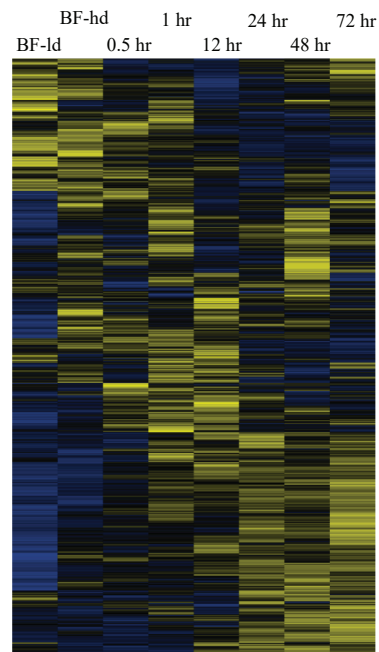**d.**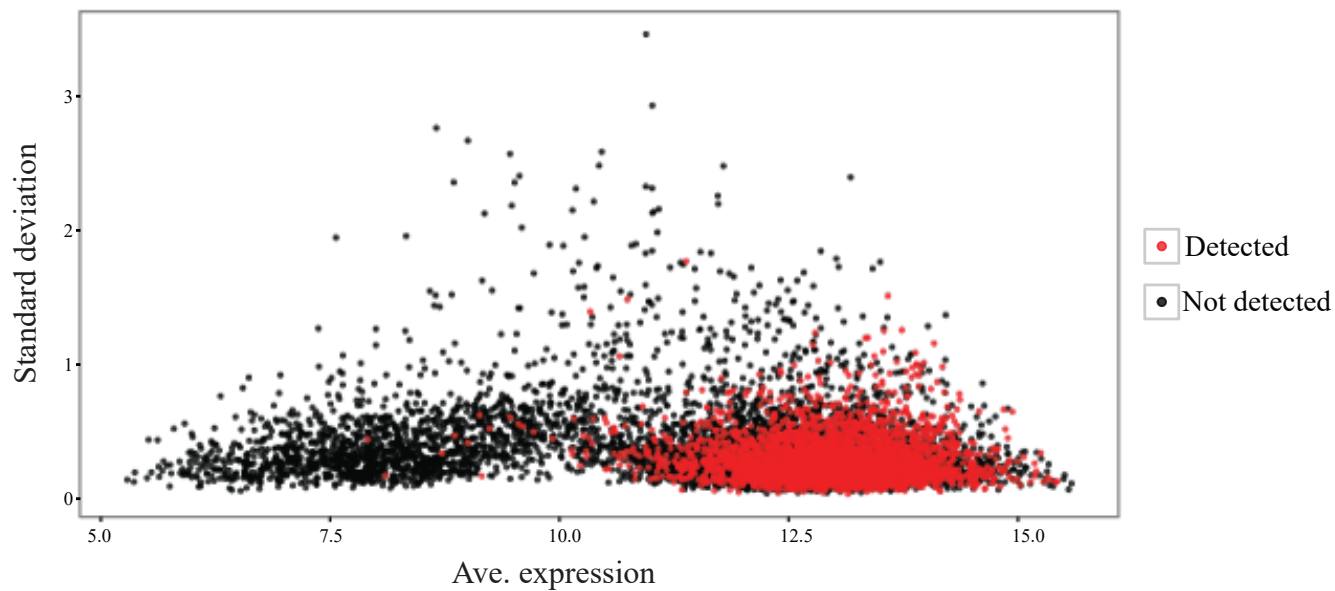

Supplement: S2 Fig — Heatmaps represent expression patterns of the identified proteins in different life stages (a, extracted from Jensen et al. [50]) and during the differentiation process from the bloodstream to the procyclic form (b, extracted from Kabani et al. [52]) and (c, extracted from Queiroz et al. [51]). For each study, the expression data of each gene was normalized to have mean zero and standard deviation equal to one. The yellow color represents up-regulation and blue indicates the down-regulation. d) Plot represents average expression of transcripts against their standard deviations in five different life stages of T. brucei [50]. As shown, proteins identified in this proteomic-based study are biased towards more abundant transcripts in the cell. (PDF) [file pntd.0004533.s002.pdf]

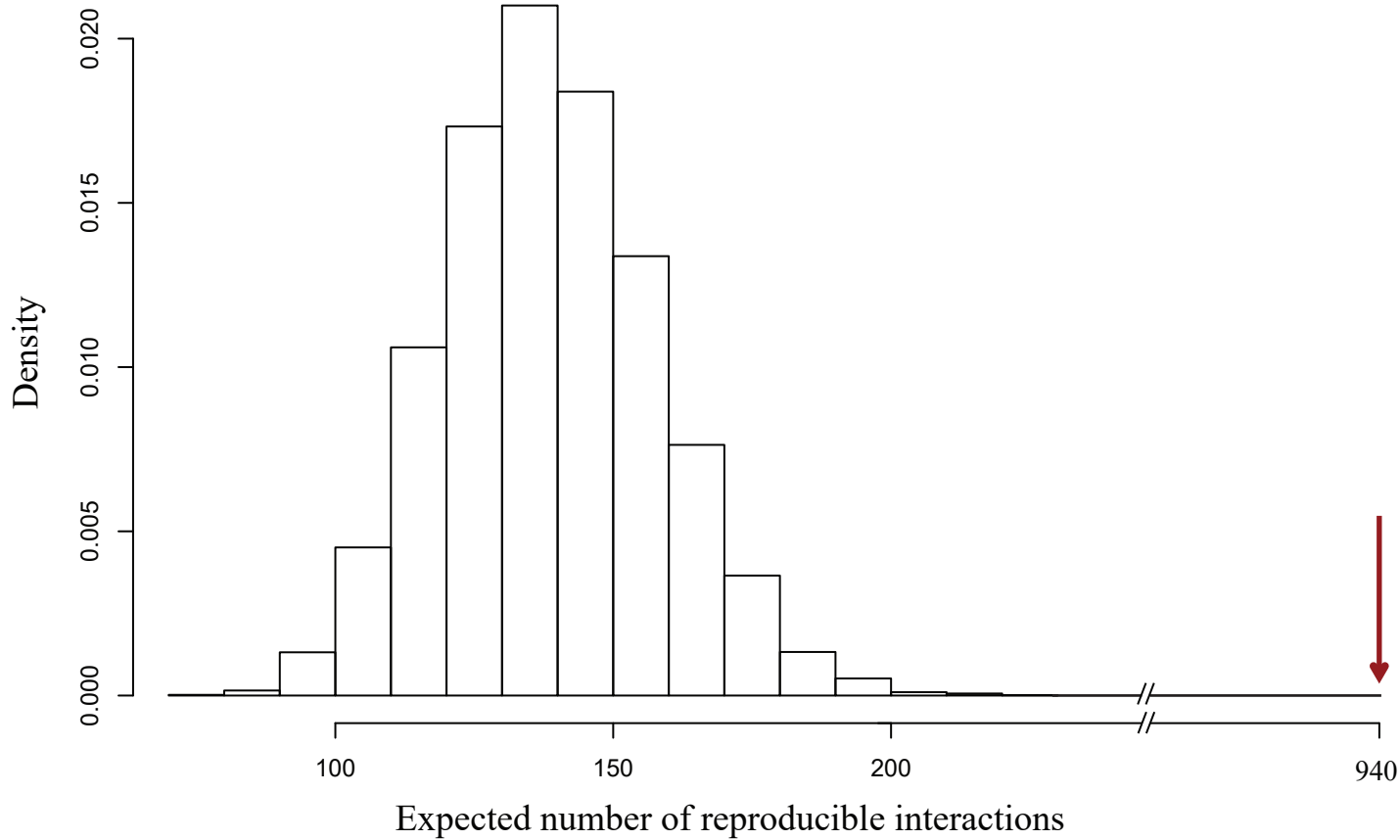

Supplement: S3 Fig — One hundred different random datasets were generated by shuffling protein labels from each of the whole cell-GG and mitochondrial-GG datasets. Applying the same analysis pipeline as TbCF net, the distribution corresponding to the number of reproducible interactions (FDR ≤0.05 in one dataset and p-value ≤0.05 in the other) between each possible combination of random datasets (10,000 combinations in total) were observed. As illustrated, the expected number of reproducible interactions by chance is 137. However, the whole cell-GG and mitochondrial-GG networks share 940 reproducible interactions (the red arrow) with each other. (PDF) [file pntd.0004533.s003.pdf]

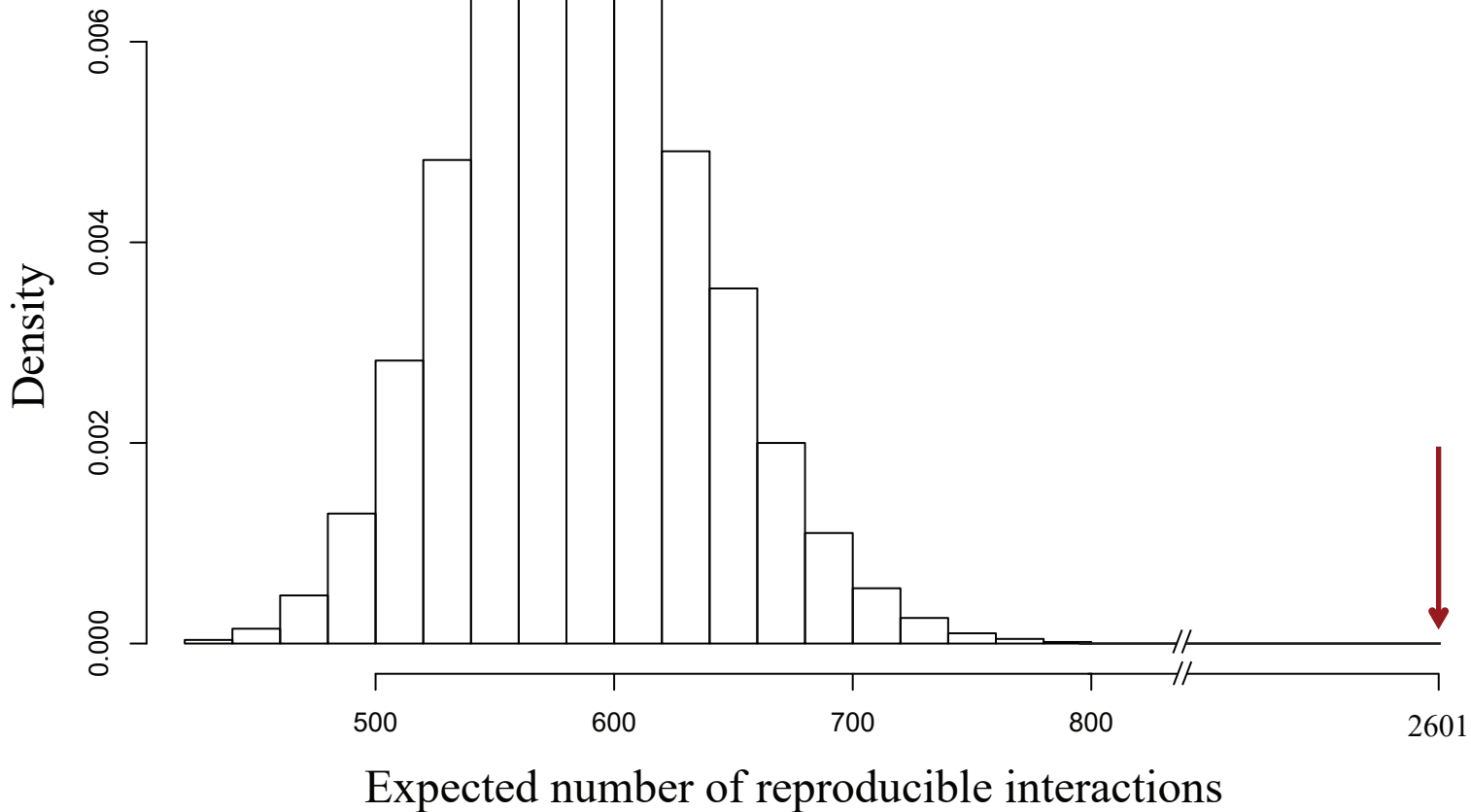

Supplement: S4 Fig — The four fractionation datasets were categorized based the employed fractionation approach to the IEX and GG groups. Within each group gene labels were randomized, while preserving the linkages between the datasets inside the group; e.g., if the gene label for geneA was shuffled to the gene99 in one dataset, the same gene was also called gene99 in the other dataset present in that particular group. This process repeated one hundred times for each group, generating one hundred random groups for each of IEX and GG groups. Networks were generated for each combination of groups applying the same criteria as those applied to construct TbCF net. Next, the distribution corresponding to the number of reproducible interactions (FDR ≤0.05 in one group and p-value ≤0.05 in the other) among each possible combination of random groups (10,000 combinations in total) were observed. As illustrated, the expected number of reproducible interactions by chance is 587. However, the GG-derived and IEX-derived networks share 2601 reproducible interactions (the red arrow) with each other. (PDF) [file pntd.0004533.s004.pdf]

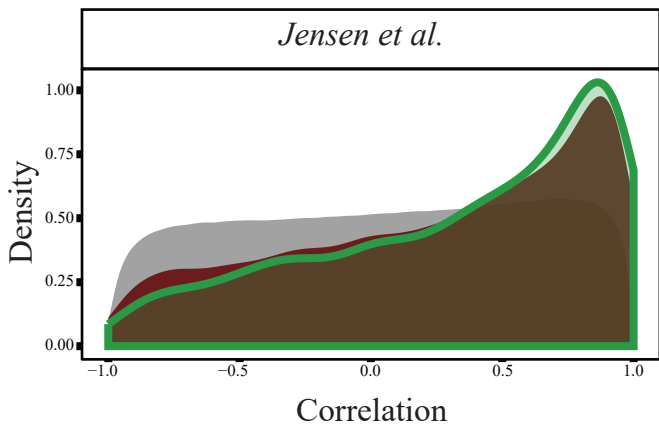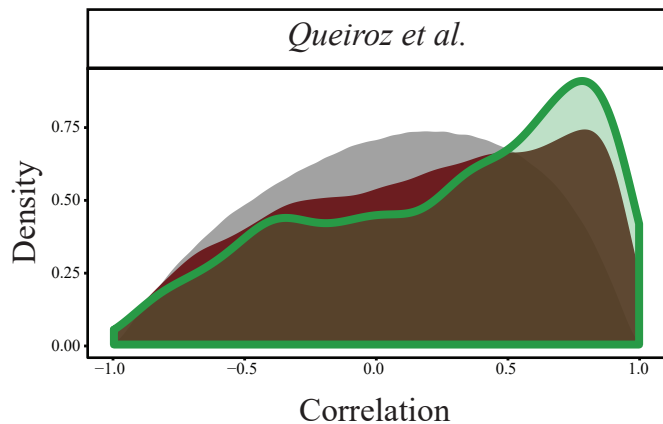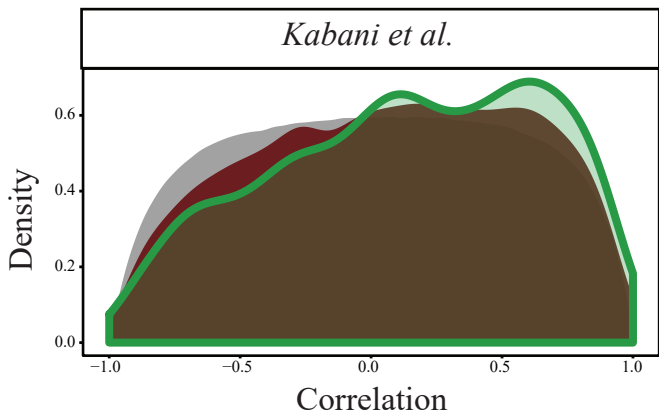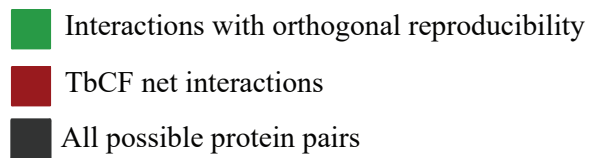

Supplement: S8 Fig — Pearson correlation coefficient was calculated between each of that interacting protein pairs in TbCF net (the red curves) and all possible pairs of the proteins identified in this study, as a control (the gray curves). Data from three datasets were used for this analysis [50–52]. As shown, the reproducible interactions (i.e., those co-fractionating in both fractionation approaches) constantly had higher similarity in terms of co-expression compared to the non-reproducible interactions. (PDF) [file pntd.0004533.s008.pdf]

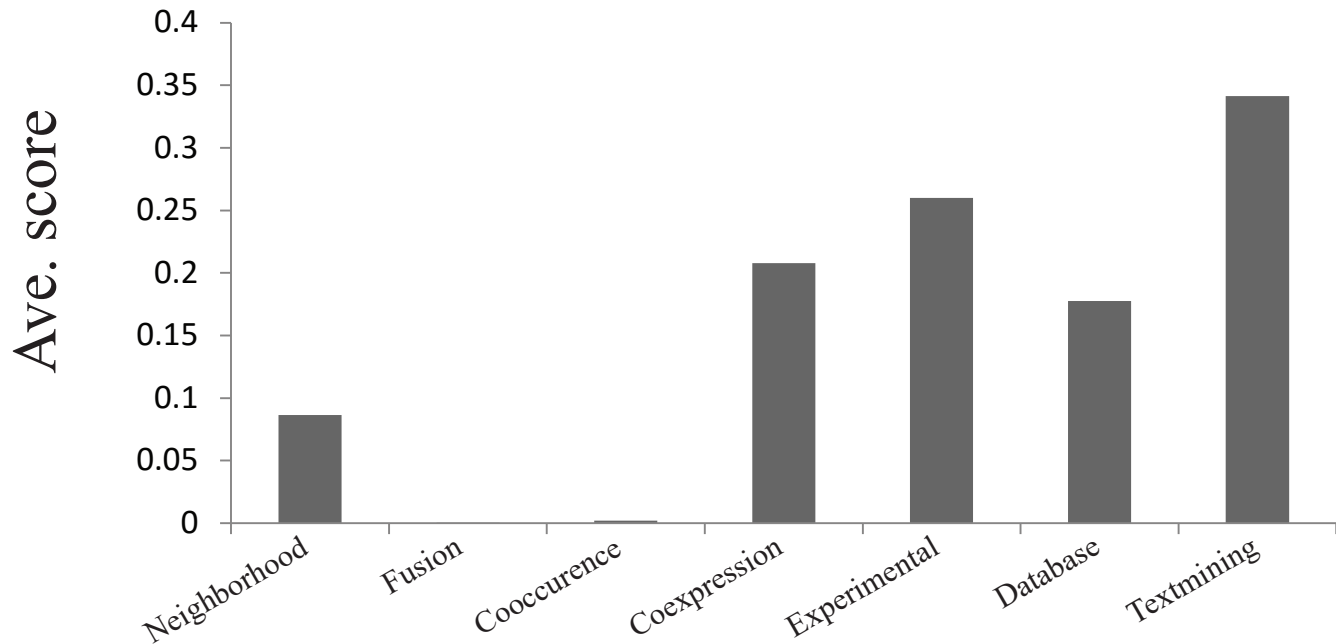

Supplement: S9 Fig — All interacting protein pairs related to T. brucei were downloaded from STRING v10 (5), and the average score for each inference method were calculated accordingly. (PDF) [file pntd.0004533.s009.pdf]

**a.**

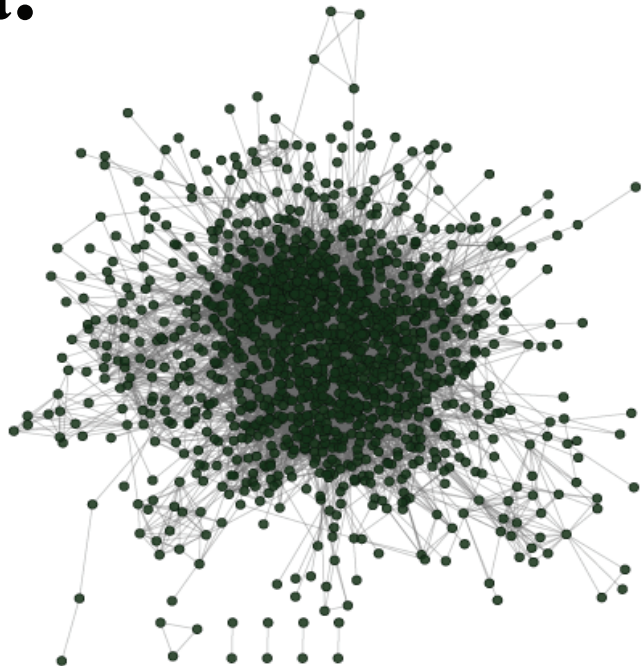

**b.**

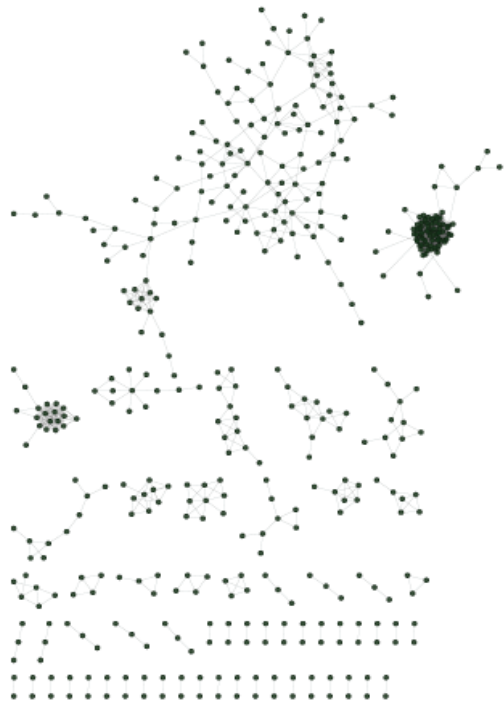

Supplement: S10 Fig — a) Structure of extracted STRING network with the medium evidence score for proteins present in TbCF net. b) Structure of TbCFSTRING network that was generated by considering interactions that are present in both TbCF net and STRING-derived network. As illustrated, the integration has led to the generation of a more modular network. (PDF) [file pntd.0004533.s010.pdf]

a.

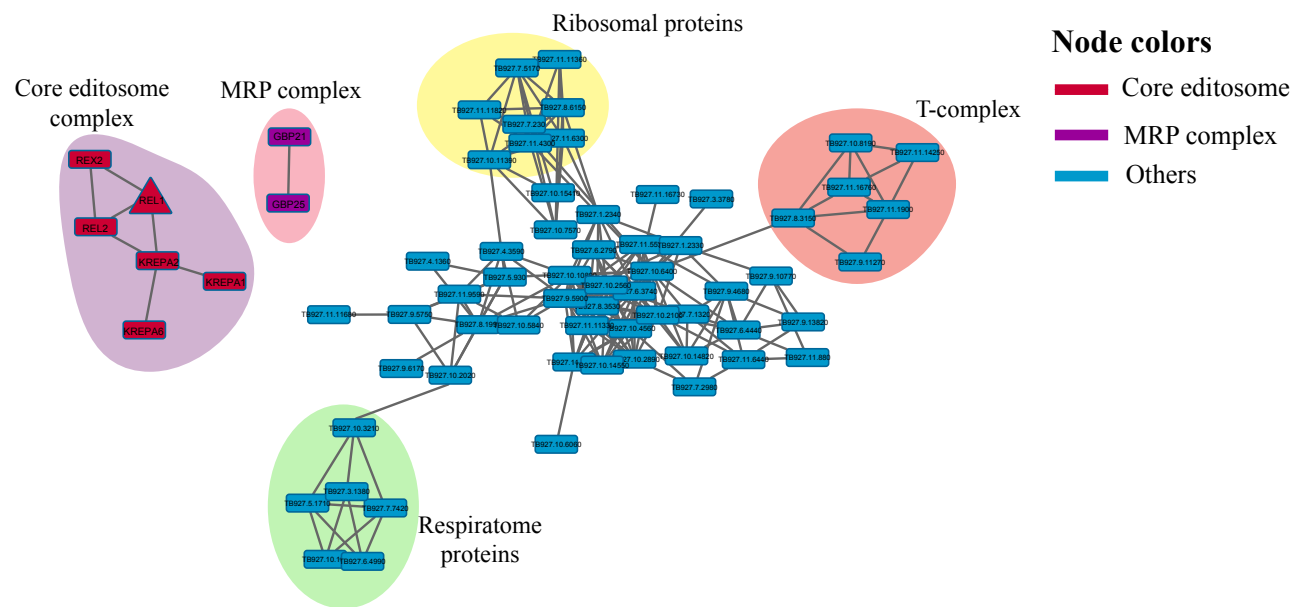

b.

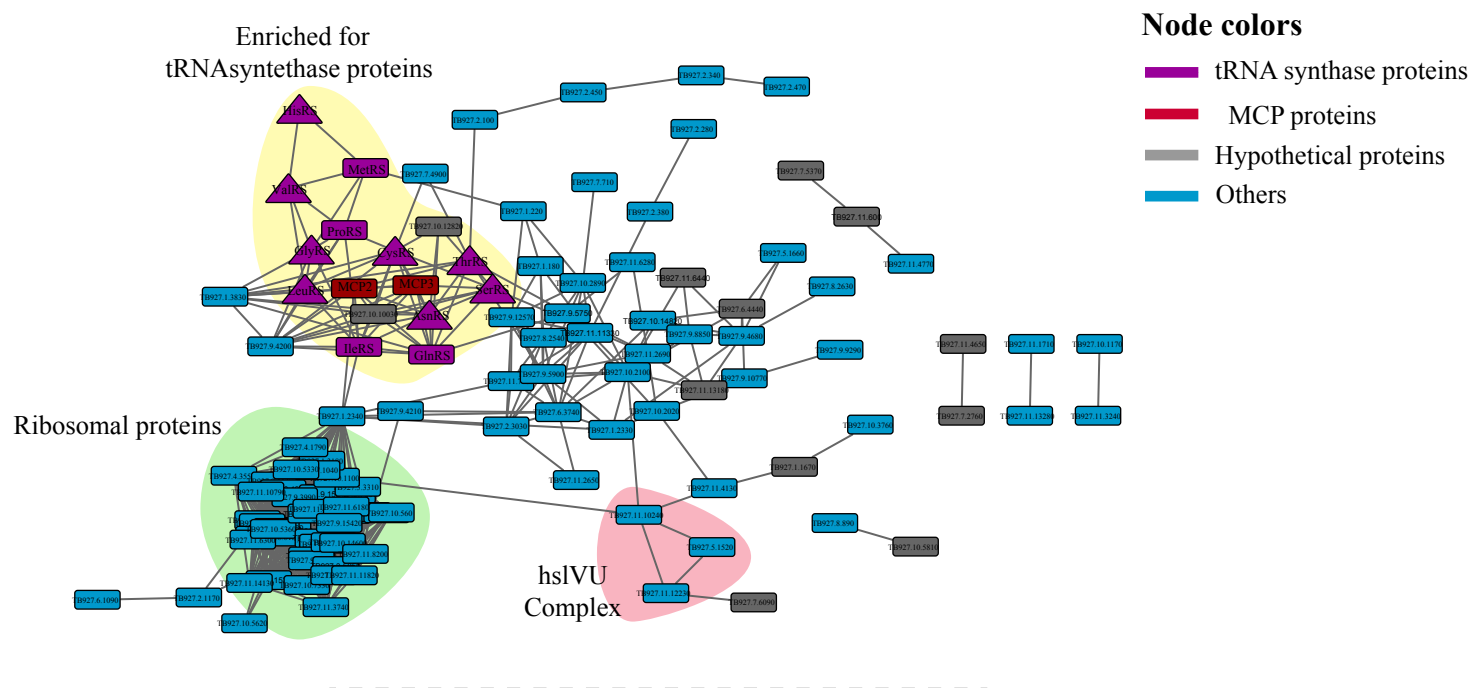

c.

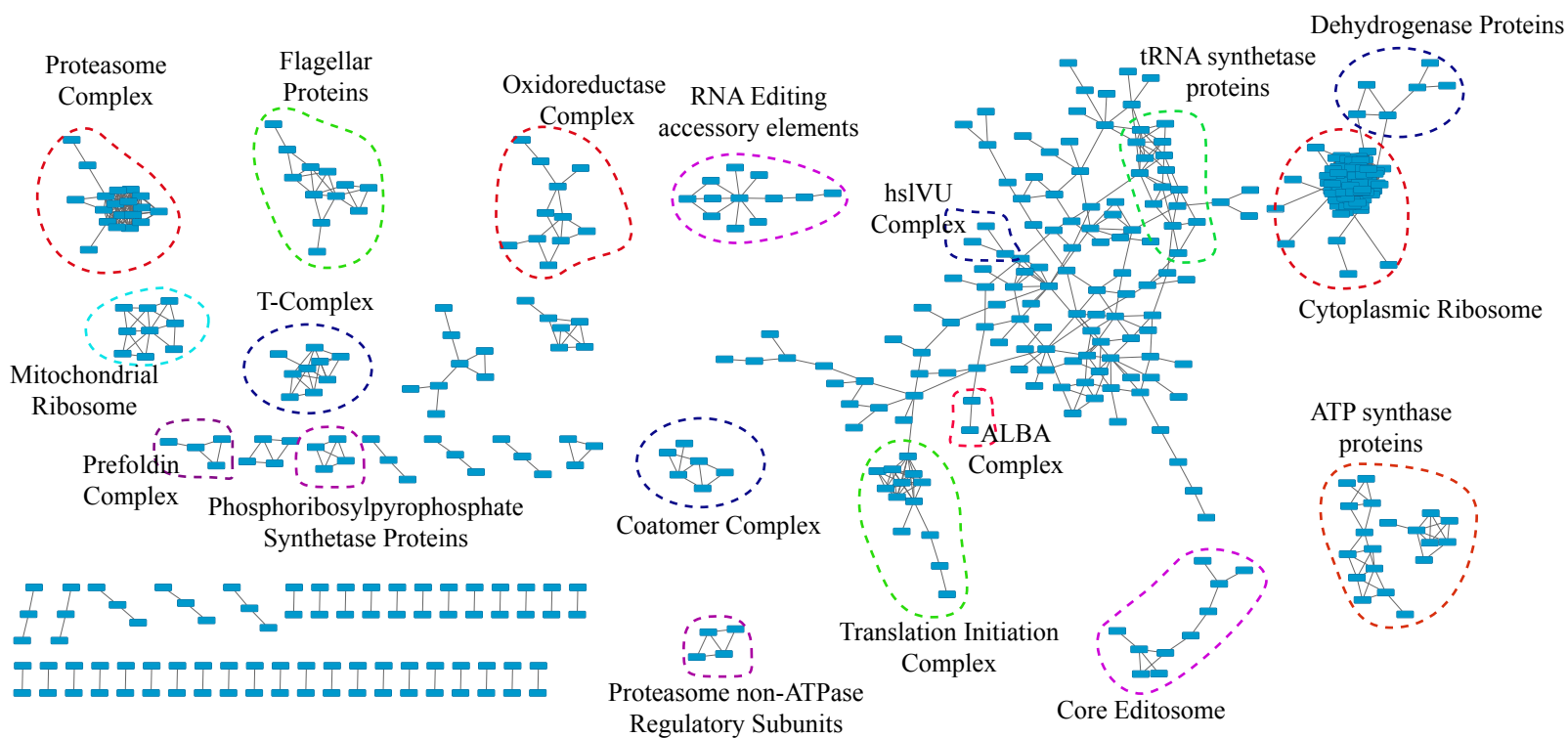

Supplement: S11 Fig — (PDF) [file pntd.0004533.s011.pdf]

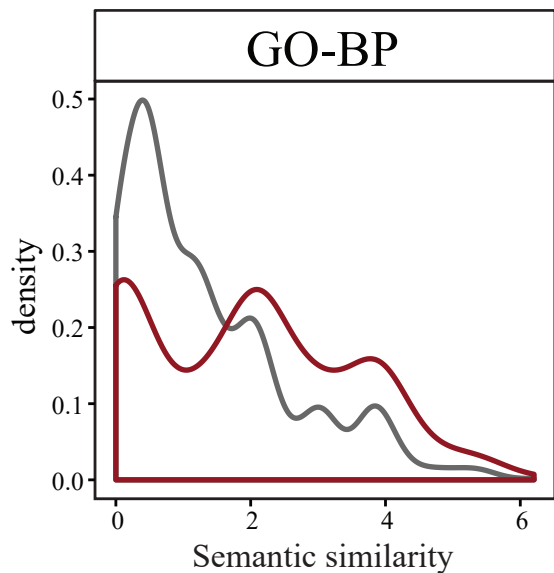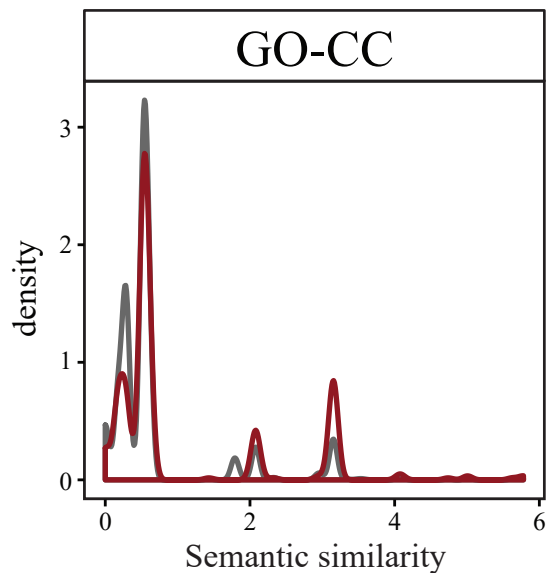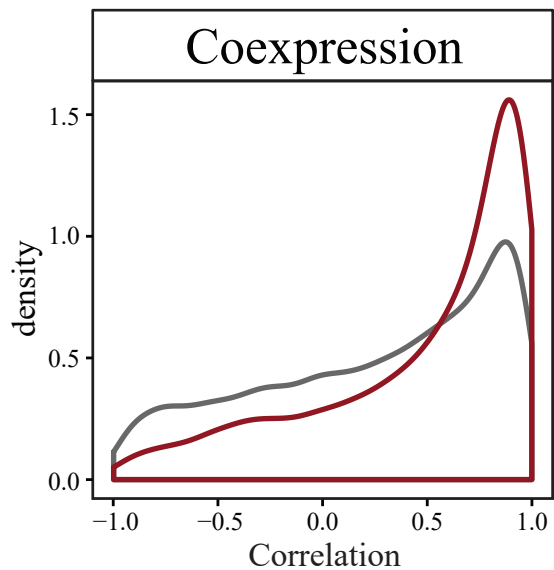

**Color key**

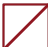 TbCF<sub>HC</sub> net

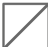 TbCF net

Supplement: S12 Fig — Comparison of GO-BP, GO-CC, and Co-expression distributions indicates TbCFHC is significantly improved over TbCF net. For co-expression analysis, data from [50] were used. (PDF) [file pntd.0004533.s012.pdf]

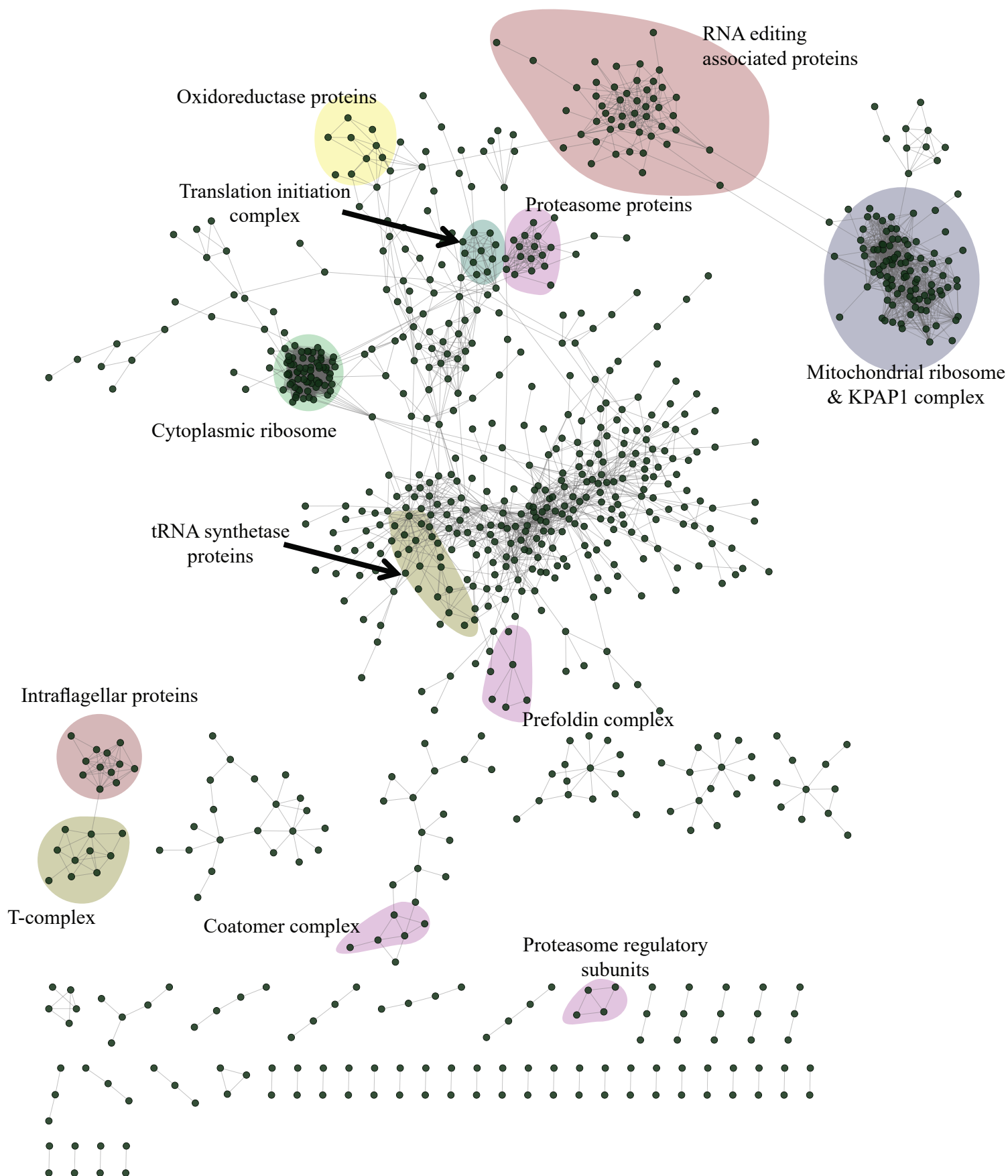

Supplement: S13 Fig — As illustrated, clustering of TbCFHC net led to the recovery of many of previously identified complexes in T. brucei. Clustering also predicted some new complexes and assigned new members to the previously characterized complexes. (PDF) [file pntd.0004533.s013.pdf]

Glycerol gradient fractionation

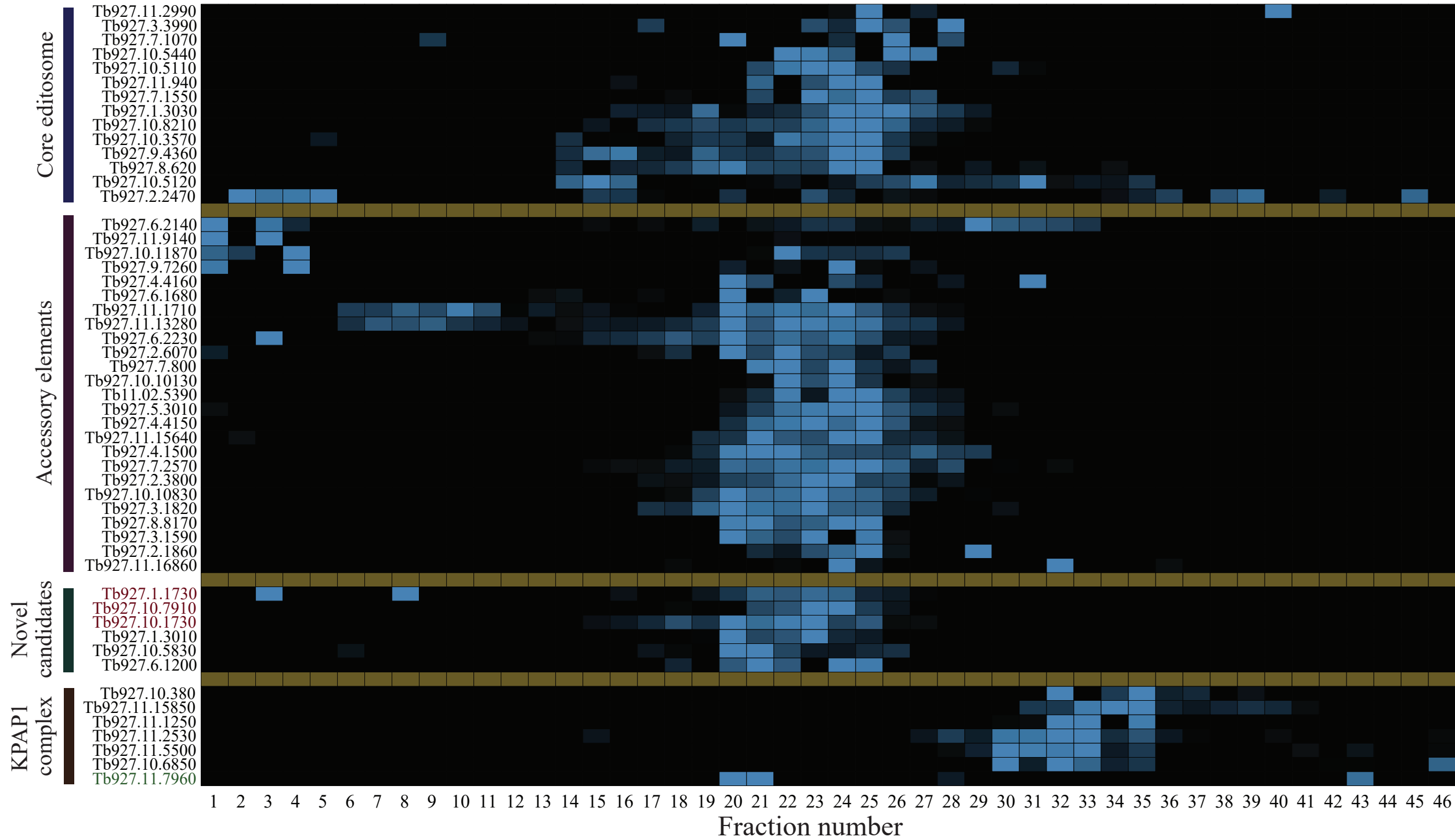

Ion exchange fractionation

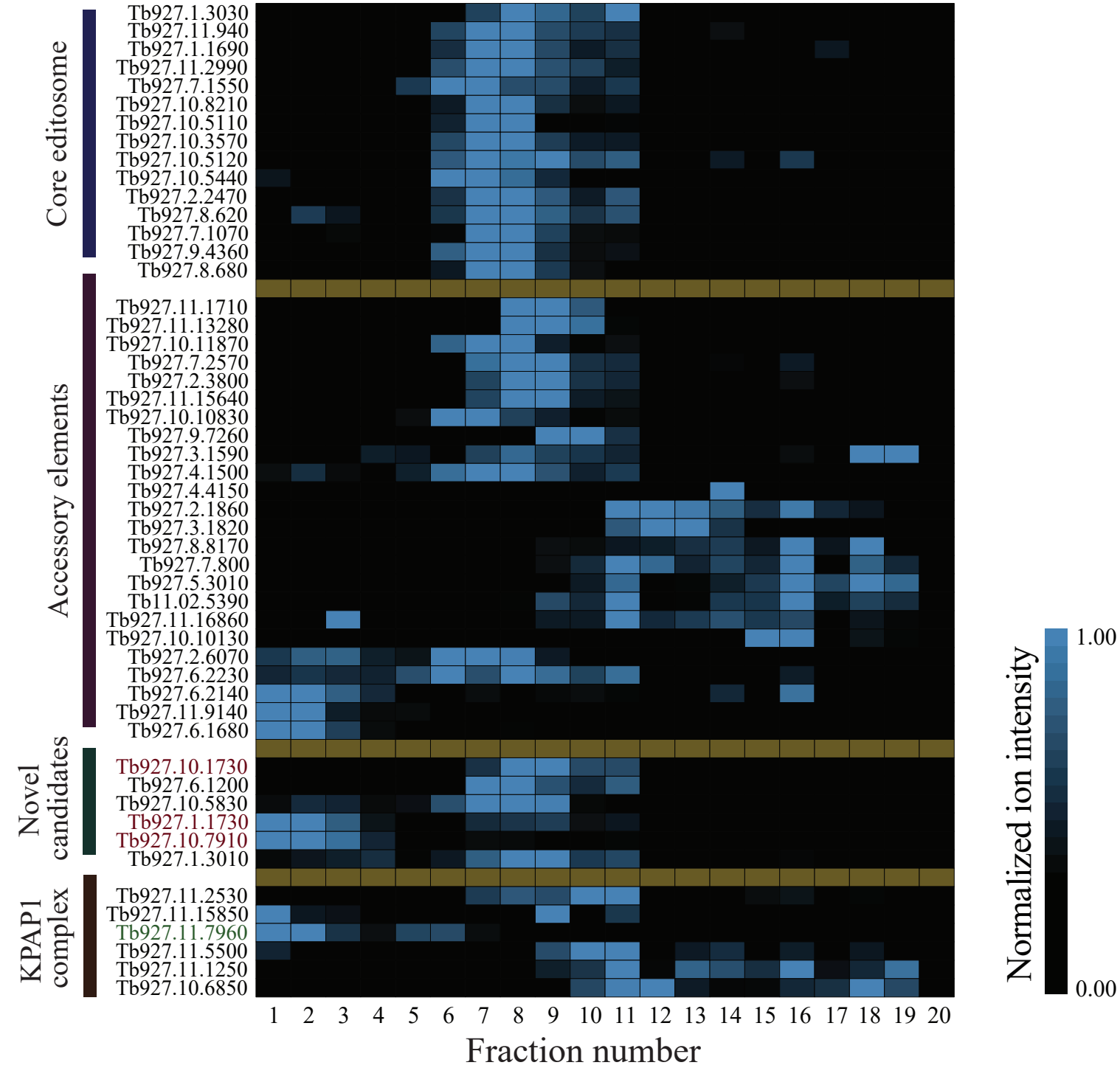

Supplement: S14 Fig — As illustrated, GG sedimentation patterns demonstrate the involvement of KPAP1 (in green color) protein with RNA-editing machinery, KPAP1 complex, and ribosomal proteins. Selected proteins for experimental validation are represented in red color. (PDF) [file pntd.0004533.s014.pdf]

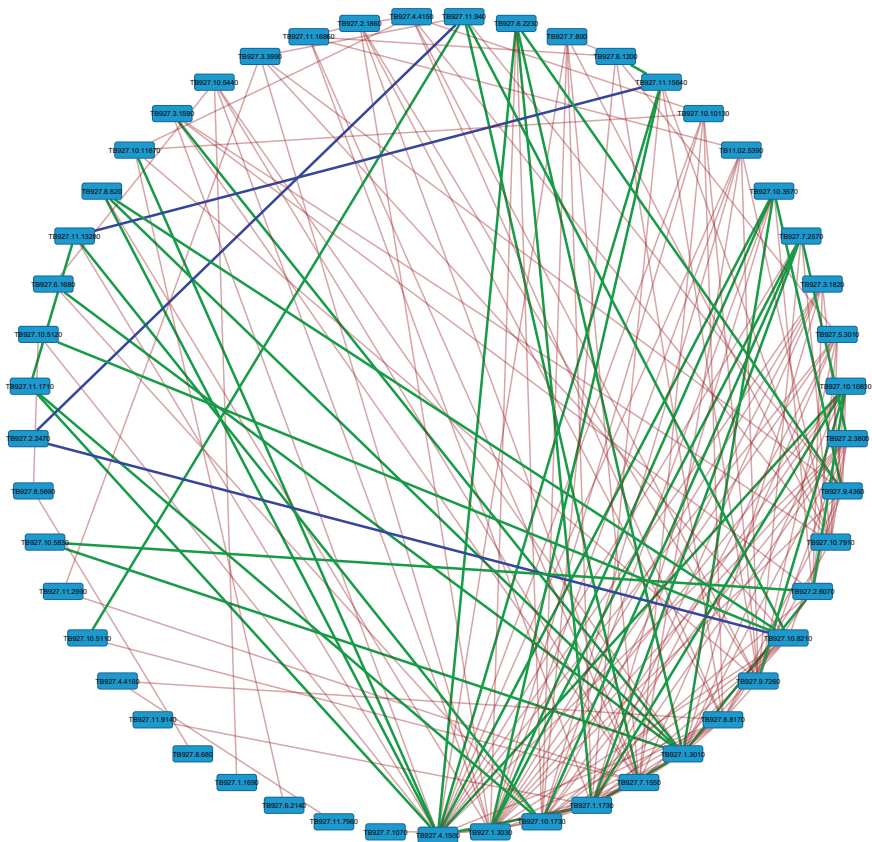

## Edge colors

- Reproducible  
— Identified by GG  
— Identified by IEX

Supplement: S15 Fig — Clustering of TbCFHC net predicted the involvement of 50 proteins in the RNA-editing machinery. This figure illustrates the interactions that were inferred based on the Mitochondrial-GG and Mitochondrial-IEX experiments. The edge color represents the source of experiment which interaction was inferred from. (PDF) [file pntd.0004533.s015.pdf]

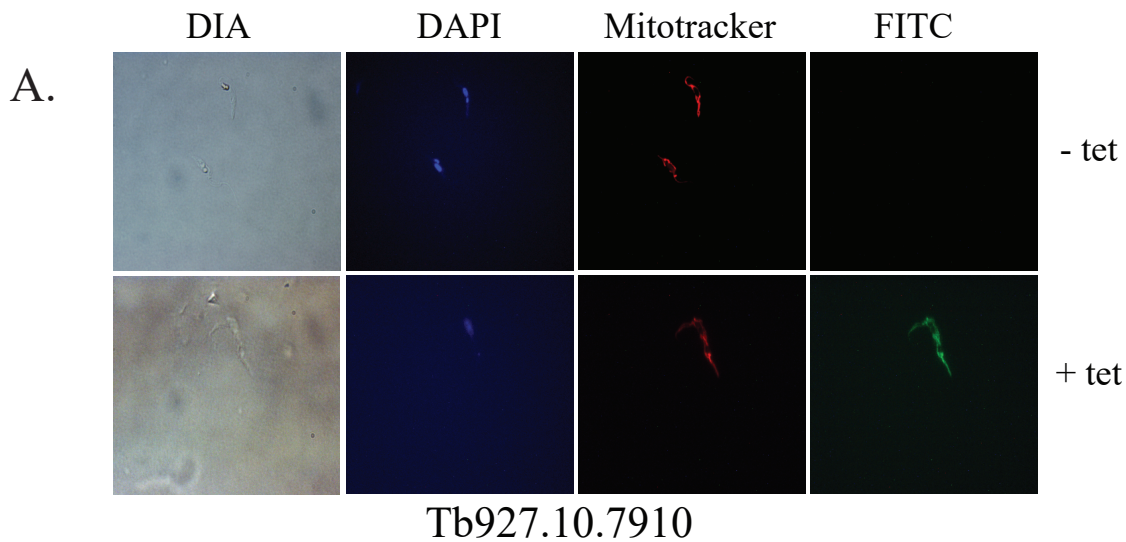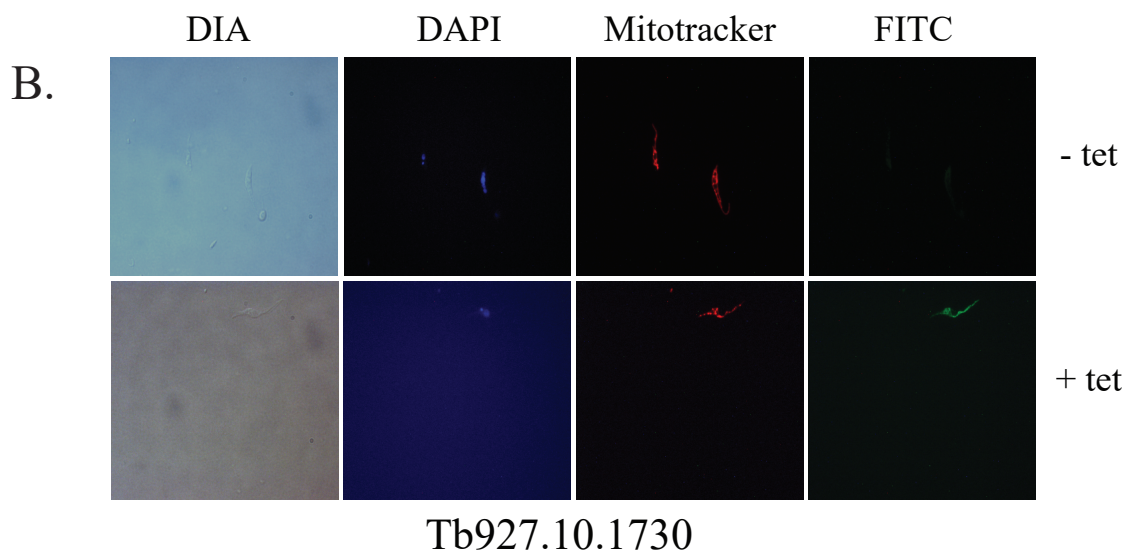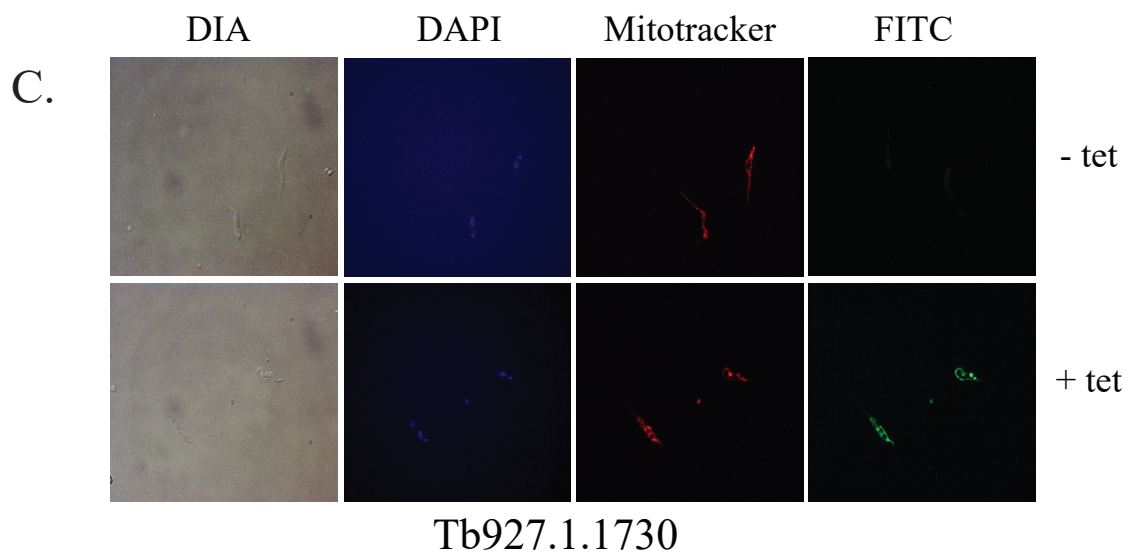

Supplement: S16 Fig — Mitochondrial localization for C-terminal 2×myc–tagged Tb927.1.1730, Tb927.10.1730, and Tb927.10.7910 proteins. Anti-myc antibody was used to detect tagged cells in procyclic life stage. Mitochondrial localization was observed for all three genes after 48 hours of induction by Tetracyclin. Mitotracker was used to stain mitochondria and DAPI to detect kinetoplasts and nuclei. FITC (fluorescence isothiocyanide) was used to dye the tagged proteins. DIA (dialkyl aminos tyryl) was used to stain the parasite. (PDF) [file pntd.0004533.s016.pdf]
