# Supplementary material for: A Protein Complex Map of Trypanosoma brucei
Source: PLoS Negl Trop Dis. 2016 Mar 18;10(3):e0004533. doi: 10.1371/journal.pntd.0004533 (PMC4798371; doi:10.1371/journal.pntd.0004533)
Supplement: S5 Fig — Interactions in each of the mitochondrial-GG and mitochondrial-IEX networks were categorized as either those occurring among proteins known to be associated with the RNA editing machinery or others. Yellow region demonstrates the area that is over-represented (p-value ≤0.05) for interactions among the proteins associated with the RNA editing machinery and blue demonstrates the regions with under-representation (p-value ≤0.05) of those interactions. Enrichment at each point on the graph was calculated using a two-tailed hypergeometric test by focusing on the closest 38 interactions to that point. (PDF) [file pntd.0004533.s005.pdf]

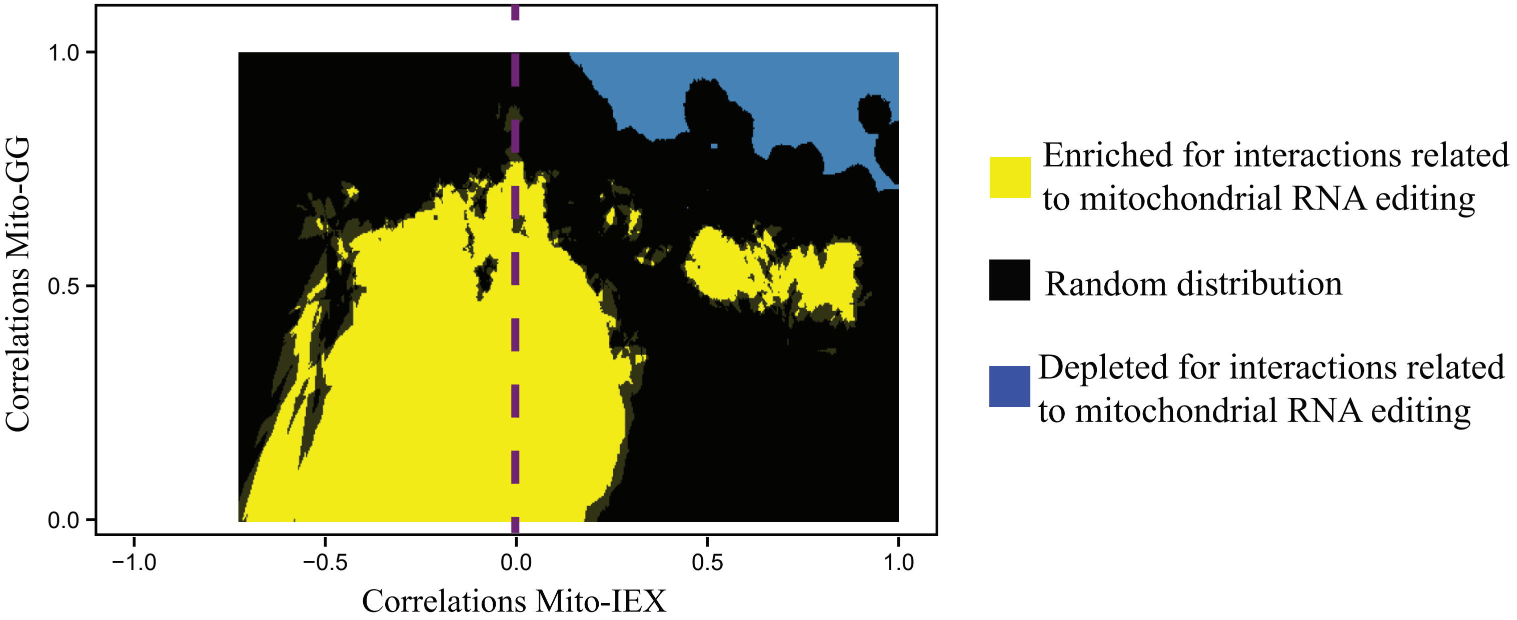

Correlations Mito-GG
1.0
0.5
0.0
−1.0
−0.5
0.0
0.5
1.0
Correlations Mito-IEX
Enriched for interactions related to mitochondrial RNA editing
Random distribution
Depleted for interactions related to mitochondrial RNA editing
