# Supplementary material for: A Protein Complex Map of Trypanosoma brucei
Source: PLoS Negl Trop Dis. 2016 Mar 18;10(3):e0004533. doi: 10.1371/journal.pntd.0004533 (PMC4798371; doi:10.1371/journal.pntd.0004533)
Supplement: S6 Fig — The average GO-BP semantic similarity was calculated across different z-score cut-off thresholds for the cytosolic-IEX and mitochondrial-IEX experiments, separately. The purple line highlights the co-elution cut-off threshold corresponding to a false discovery rate of 0.05. To examine the applied filtration steps (elimination of noise sensitive, unshared-peak, early sedimenting, and non-reproducible interactions), we applied the same analysis to the cytosolic-IEX network before and after the filtration steps, but not the mitochondrial-IEX network because of its small size. As shown, the employed filtration steps have led to an increase in precision. However, the reproducible interactions constantly had higher similarity compared to the non-reproducible interactions. (PDF) [file pntd.0004533.s006.pdf]

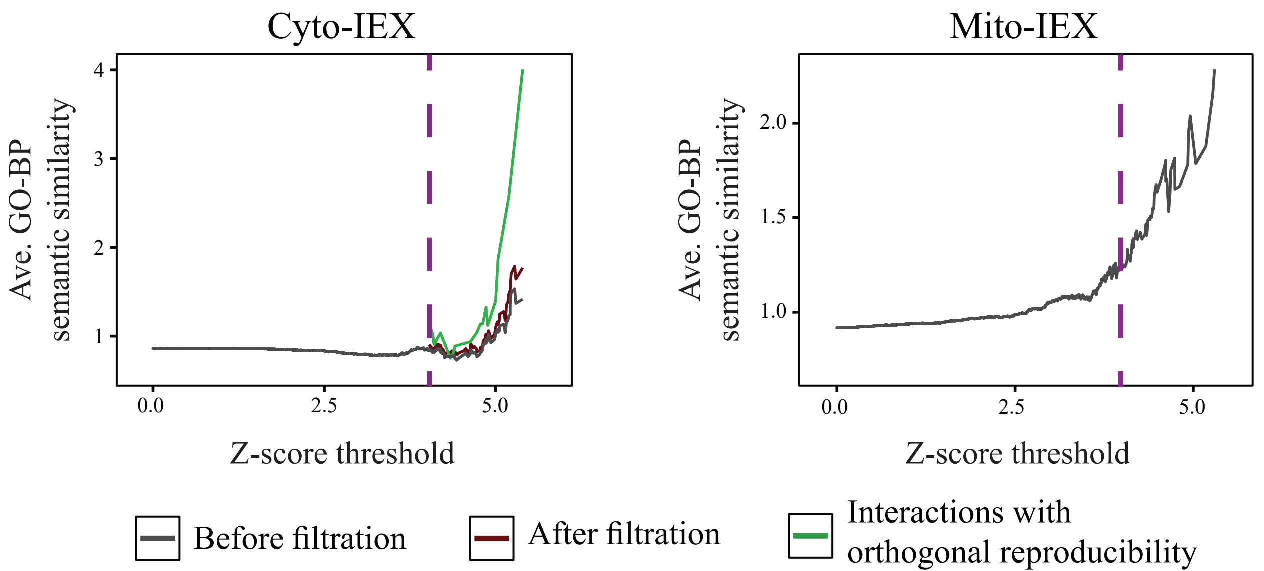
Cyto-IEX
Mito-IEX
Ave. GO-BP semantic similarity
4
3
2
1
2.0
1.5
1.0
0.0
2.5
5.0
Z-score threshold
Before filtration
After filtration
Interactions with orthogonal reproducibility
