# Supplementary material for: A Protein Complex Map of Trypanosoma brucei
Source: PLoS Negl Trop Dis. 2016 Mar 18;10(3):e0004533. doi: 10.1371/journal.pntd.0004533 (PMC4798371; doi:10.1371/journal.pntd.0004533)
Supplement: S7 Fig — The percentage of protein pairs with a shared KEGG attribute was calculated across different zscore cut-off thresholds for all four fractionation datasets. (PDF) [file pntd.0004533.s007.pdf]

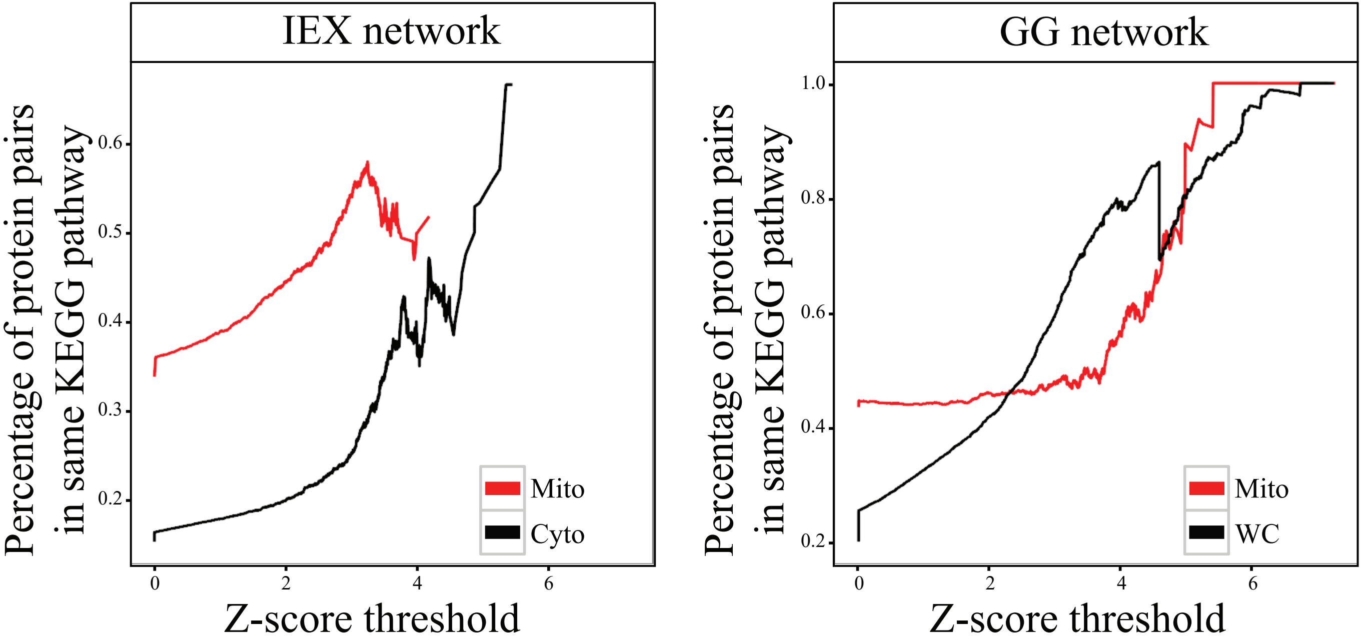
IEX network
GG network
Percentage of protein pairs in same KEGG pathway
Z-score threshold
0.2
0.3
0.4
0.5
0.6
0.8
1.0
0
2
4
6
Mito
Cyto
WC
