# Supplementary material for: A Protein Complex Map of Trypanosoma brucei
Source: PLoS Negl Trop Dis. 2016 Mar 18;10(3):e0004533. doi: 10.1371/journal.pntd.0004533 (PMC4798371; doi:10.1371/journal.pntd.0004533)
Supplement: S1 Table — (DOCX) [file pntd.0004533.s017.docx]

**Table S1. Oligonucleotides used in this study.**

qRT-PCR primers

| Name | 5’ primer | 3’ primer |
| --- | --- | --- |
| COIII pre | GAAACCAGATGAGATTGTTTGCA | TTCATTCCAACTAAACCCTTTCC |
| COIII edit | TTGTGTTTTATTACGTTGTATCCAGTATTG | CGAAAGCAAACTCACAACACAAA |
| CYbpre | ATATAAAAGCGGAGAAAAAAGAAAG | CCCATATATTCTATATAAACAACCTGACA |
| CYbedit | AAATATGTTTCGTTGTAGATTTTTATTATTT | CCCATATATTCTATATAAACAACCTGACA |
| A6 pre | TTGCCTTTGCCAAACTTTTAGAAG | ATTCTATAACTCCAAAATCACAACTTTCC |
| A6 edit | GATTTATTTTGGTTGCGTTTGTTATTATG | CAAACCAACAAACAAATACAAATCAAAC |
| CoIIpre | ATTACAGTGTAACCATGTATTGACATT | TTCATTACACCTACCAGGTTCTCT |
| CoIIedit | ATTACAGTGTAACCATGTATTGACATT | ATTTCATTACACCTACCAGGTATACAA |
| Murf2 pre | GATTTTAAGATTGGCTTTGATTGA | AATATAAAATCTAGATCAAACCATCACA |
| Murf2 edit | GATTTTAATGTTTGGTTGTTTTAATTTAG | AATATAAAATCTAGATCAAACCATCACA |
| RPS12 pre | CGACGGAGAGCTTCTTTTGAATA | CCCCCCACCCAAATCTTT |
| RPS12 edit | CGTATGTGATTTTTGTATGGTTGTTG | ACACGTCGGTTACCGGAACT |
| CoI | CCCGATATGGTATTTCCTCGTATAAA | CCCCCATACCCTCTTCAGTCA |
| ND4 | CAATCTGACCATTCCATGTGTGA | TTTCAGCACAATACTTGCTAATAAAACA |
| A6-Cybprecursor | TCCGCCCAAAATTCCTCTTT | CCAATATGAATGGAATTACAATACTGAGT |
| 9S/ND8 precursor | AAAAGGTATTGTTGCCACCAA | CAACCAAAACTTAAAATTATTAAATTGATTC |
| RPS12/ND5 | GGGAACCCTTTGTTTTGGTTAAAG | TTCCTACCAAACATAAATGAACCTGAT |
| 18SrRNA | CGGAATGGCACCACAAGAC | TGGTAAAGTTCCCCGTGTTGA |
| Tb927.10.7910 | GCTTAGTCTTGCGGGTCTTG | CGCAGTAATACGCTGGAAAACAT |

**Plasmid Construction primers**

P2T7-177 plasmid

| Name | 5’ primer | 3’ primer |
| --- | --- | --- |
| Tb927.10.7910 | TAATC**GGATCC**ATGACGGCCGTTTATTATGC | TAATC**CTCGAG**ATGATTACCTCAGCTTGCCG |

C-myc plasmids

| Name | 5’ primer | 3’ primer |
| --- | --- | --- |
| Tb927.1.1730 | ATAT**AAGCTT**ATGTTGCGCTACACCA | ATAT**GTTAAC**AGCTGGAGCTCCTACTTTAT |
| Tb927.10.1730 | ATAT**AAGCTT**ATGTGGCGTTGCTCTACTC | ATAT**GGATCC**ACTTTTCCCCACAGTT |
| Tb927.10.7910 | ATTA**AAGCTT**ATGTTTTCCAGCGTATTACT | ATAT**GGATCC**CTTCCATACAACCGTTCCC |
